# Supplementary material for: Regioselective Synthesis of Potential Non-Quinonoid Prodrugs of Plasmodione: Antiparasitic Properties Against Two Hemoglobin-Feeding Parasites and Drug Metabolism Studies
Source: Molecules. 2024 Nov 7;29(22):5268. doi: 10.3390/molecules29225268 (PMC11596610; doi:10.3390/molecules29225268)

Supplementary Materials

Regioselective Synthesis of Potential Non-Quinonoid Prodrugs of Plasmodione: Antiparasitic Properties Against Two Hemoglobin-Feeding Parasites and Drug Metabolism Studies

Elena Cesar-Rodo [1], Baptiste Dupouy [1], Cécile Häberli [2], Jean-Marc Strub [3], David L. Williams [4], Pascal Mäser [2,5], Matthias Rottmann [2], Jennifer Keiser [2,5], Don Antoine Lanfranchi [1,*] and Elisabeth Davioud-Charvet [1,*]

[1] Laboratoire d'Innovation Moléculaire et Applications (LIMA), Team Bio(IN)organic & Medicinal Chemistry, UMR7042 CNRS-Université de Strasbourg-Université Haute-Alsace, European School of Chemistry, Polymers and Materials (ECPM), 25, Rue Becquerel, F-67087 Strasbourg, France;

[2] Swiss Tropical and Public Health Institute, Kreuzstrasse 2, CH-4123 Allschwil, Switzerland; pascal.maeser@swisstph.ch (P.M.); matthias.rottmann@swisstph.ch (M.R.); jennifer.keiser@swisstph.ch (J.K.)

[3] Laboratoire de Spectrométrie de Masse BioOrganique (LSMBO), IPHC UMR 7178 CNRS, Université de Strasbourg, F-67087 Strasbourg, France

[4] Department of Microbial Pathogens and Immunity, Rush University Medical Center, 1735 West Harrison Street, Chicago, IL 60612, USA; david_williams@rush.edu

[5] University of Basel, Petersgraben 1, CH-4001 Basel, Switzerland

* Correspondence: lanfranchi@unistra.fr (D.A.L.); elisabeth.davioud@unistra.fr (E.D.-C.)

Content: Pages S2-S3: Investigations on ψ-menadione post-functionalization (Scheme S1) and screening of Diels-Alder cycloaddition conditions in reactions using 3-benzylbenzoquinone **17** (Table S1). Pages S4-S7: Metabolic Studies by LC-MS analyses (Figures S1-S3). S8-S23: NMR spectra of key/new compounds.

Investigations on ψ-menadione post-functionalization: Attemps to synthesize the ψ-Plasmodiones 1 and 2 from ψ-menadione 3 and 13.

We initially synthetized the 4a-ψ-menadione **3** to attempt post-functionalization towards the synthesis of the 4a-ψ-plasmodione **1**, bearing in mind that the radical Kochi-Anderson benzylation reaction was unlikely to proceed because the ψ-menadione scaffold is not a quinone [44]. With both ψ-menadiones **3** and **13** in hands, we tested the Kochi-Anderson reaction, and as expected, the reaction did not proceed to the desired product (Scheme S1, Route A). Only a degradation of the starting material was obtained. Another possibility could be to reduce and protect the ψ-menadione core before a chloromethylation followed by a Suzuki reaction and finally a deprotection to release the desired ψ-plasmodiones (Scheme S1, Route B). However, the protection of 8a-ψ-menadione **13** led again to a degradation of the starting material during the first reduction step.

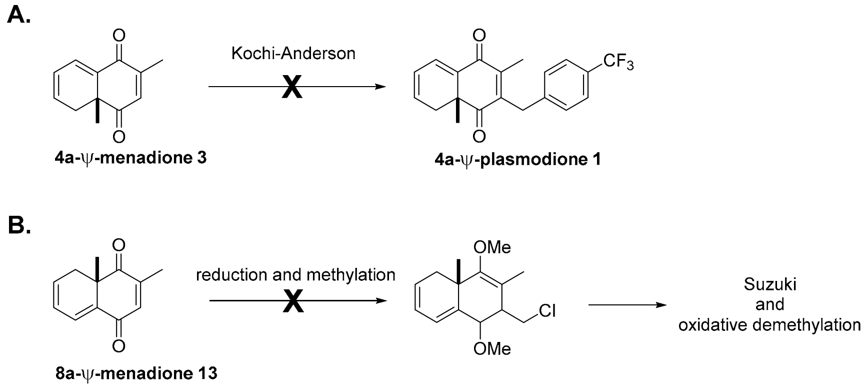

Scheme S1. Attemps to post-functionnalize the ψ-menadiones **3** and **13**.

In conclusion, because our attempts to post-functionalize the ψ-menadiones failed, we developed a strategy for the straightforward synthesis of both ψ-plasmodione regioisomers through Diels-Alder cycloadditions from a benzylated quinone precursor and a diene.

Screening of the Diels-Alder cycloaddition conditions in reactions using 3-benzylbenzoquinones

Alternatively, we performed the Diels-Alder cycloaddition directly on commercially available benzoquinones under specific conditions.

Based on the results of Lanfranchi & Hanquet, 2006 we tried to reproduce the enantioselective cycloaddition methodology from our sulfinyl-benzoquinone **17**. Using various dienes, we were not able to obtain any cycloadduct using the described conditions (entries 1-3) (Table S1). As discussed in the core of the publication, we can anticipate the benzyl chain takes places on the opposite face occupied by the *p*-tolsyl chain creating steric hindrance on both sites of the dienophile.

Table S1. Diels-Alder cycloaddition reaction with sulfinylquinone **17** with various dienophiles.

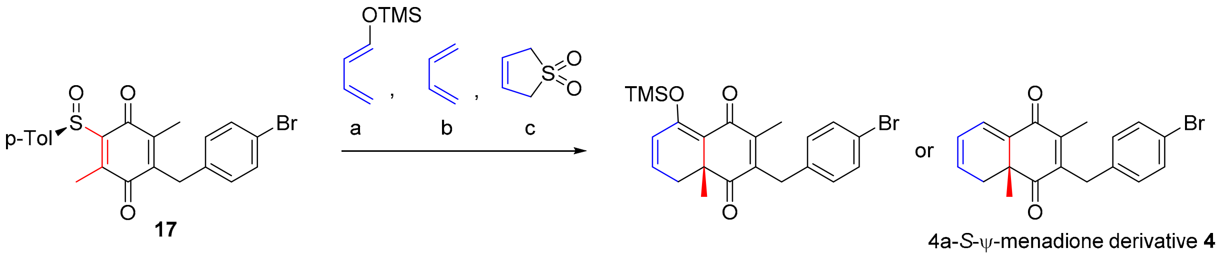

Entry	Diene	Catalyst	Solvent	Condition	Yield [%]
1	a (1.1 equiv.)	-	CH_2Cl_2	25 °C, 12 h	degradation
2	b (excess)	$ZnBr_2$ (1.2 equiv.)	CH_2Cl_2	-40 °C, 6 h then -15 °C for 4 days	s.m.
3	c (1.1 equiv.)	-	CH_2Cl_2	125 °C, 2 h 30 min in a sealed tube	degradation

s.m. means starting material.

Metabolic Studies

Calibration: plasmodione (PD) at 1, 2, 5, and 10 µM

A

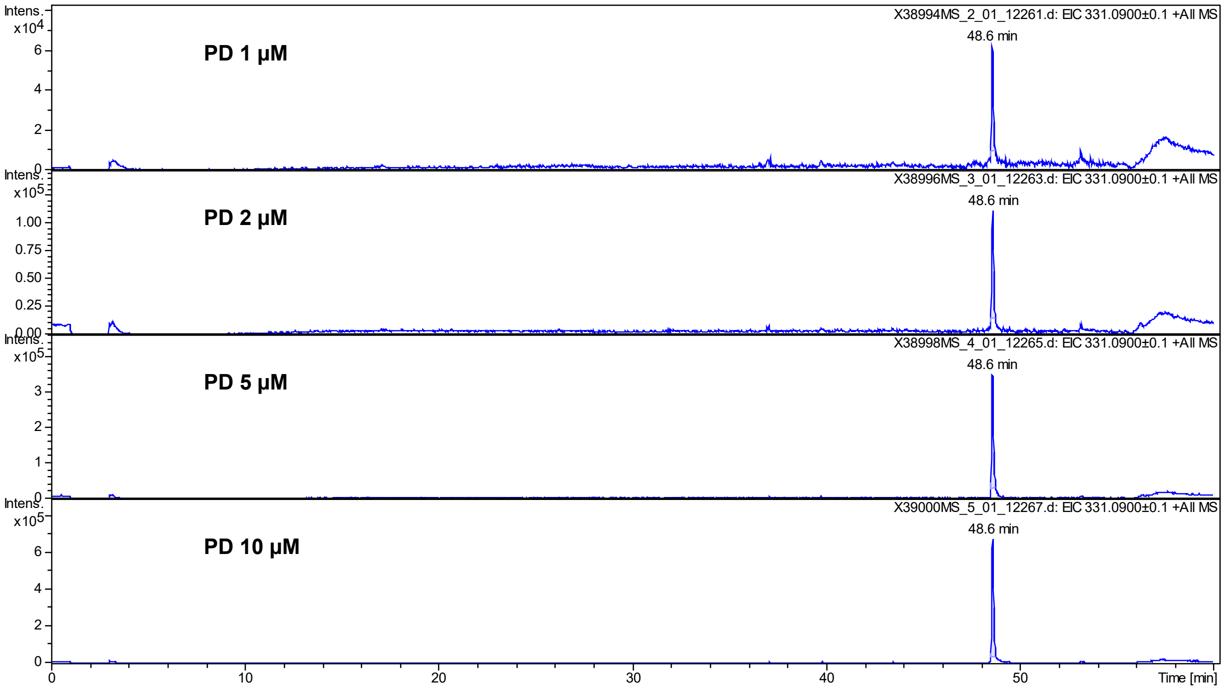

B

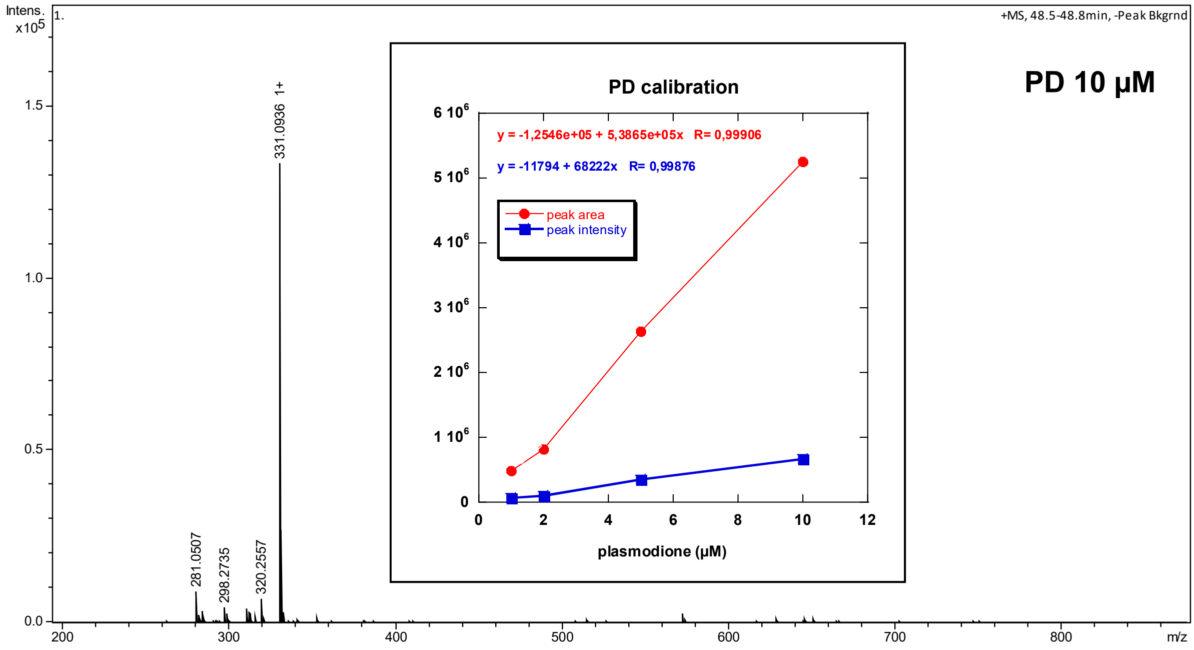

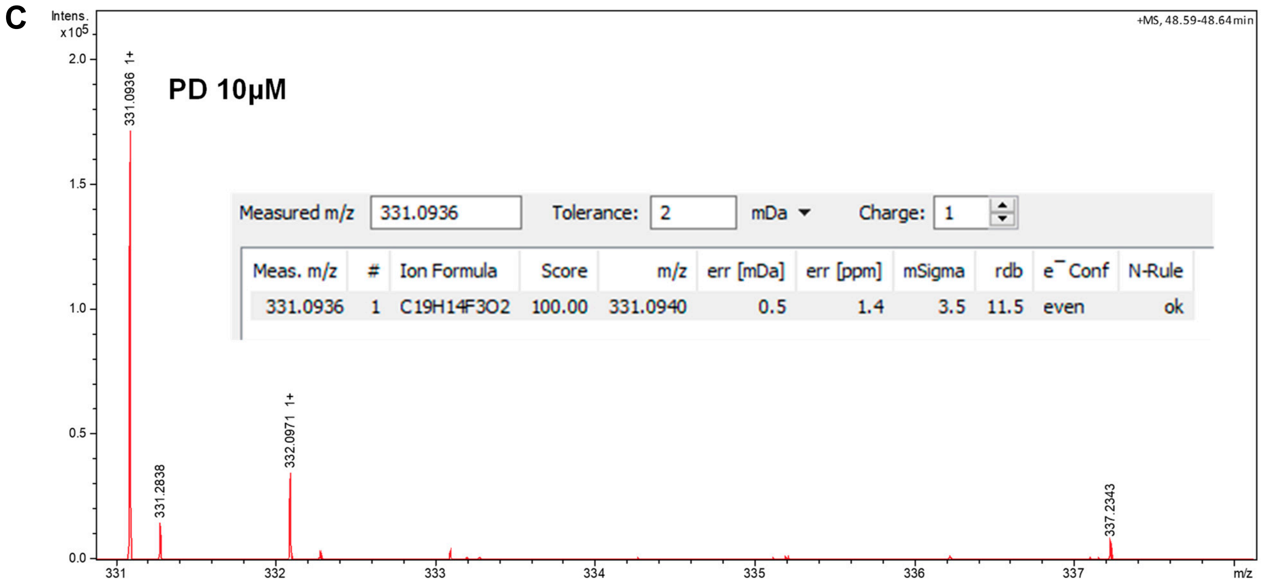

Figure S1. Plasmodione Calibration curves. Panel A: Extracted ion chromatograms (EICs) corresponding to the mass of **PD** (RT = 48.6 min) after LC-MS analysis of PD solutions with known concentrations: 1, 2, 5, and 10 µM. Panel B: PD calibration curves expressing peak area or peak intensity as a function of PD concentration. Panel C: Extracted ion chromatogram after LC-MS analysis of 10 µM PD solution showing both the observed *m/z* of **PD** (331.0936, RT = 48.6 min) and the calculated exact mass of **PD** (*m/z:* 331.0940, error: 1.4 ppm).

LC-MS analysis of reaction mixtures:

MS analysis of the EIC peak at RT = 46.2 min

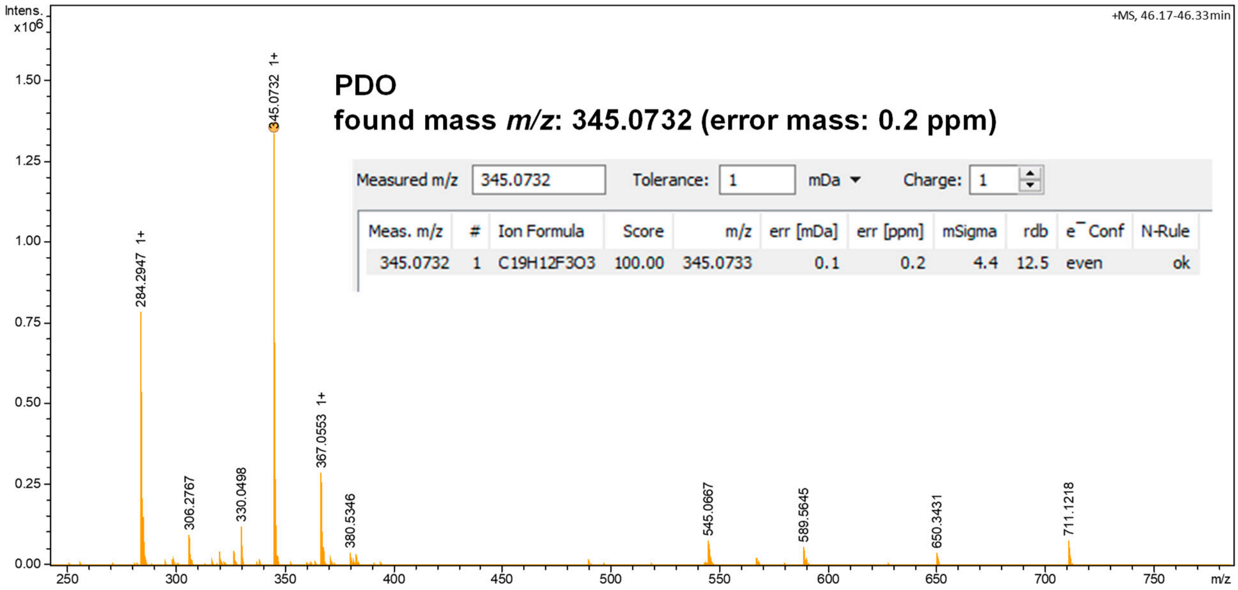

Figure S2. Extracted ion chromatograms (EIC) after LC-MS analysis of 10 µM PDO solution showing both the observed mass of **PDO** (RT = 46.2 min, *m/z*: 345.0732) and the calculated exact *m/z* of **PDO** (345.0733, error: 0.2 ppm).

A

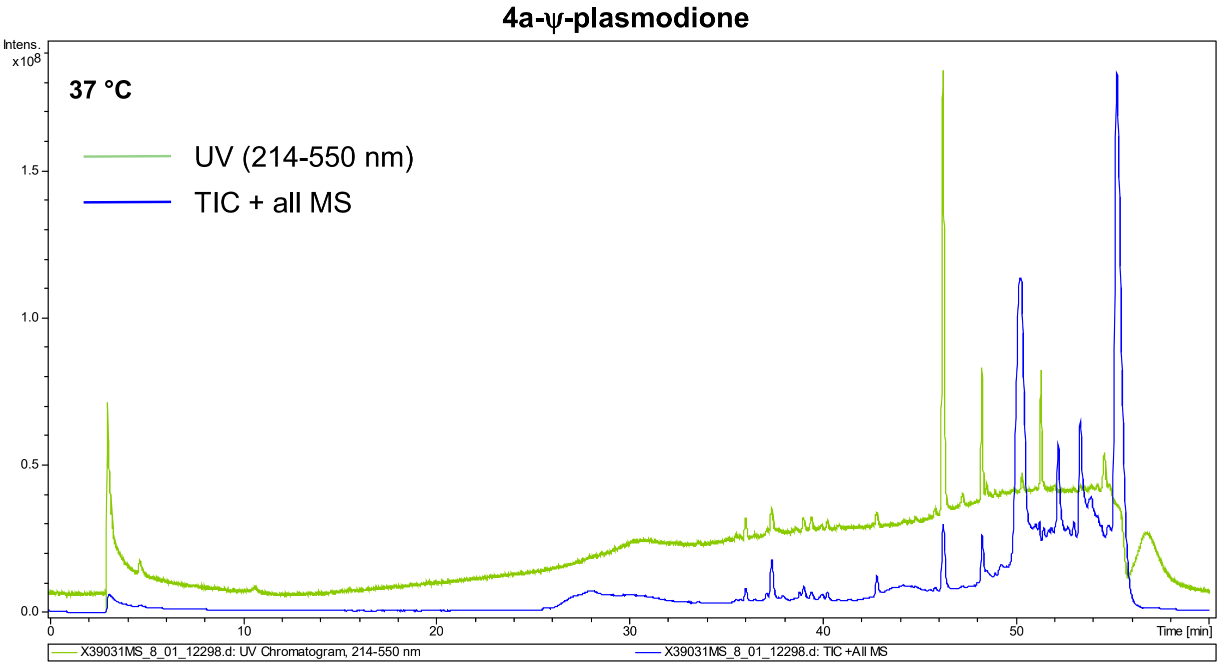
4a-ψ-plasmodione
Intens.
x10^8
37 °C
UV (214-550 nm)
TIC + all MS
1.5
1.0
0.5
0.0
0
10
20
30
40
50
Time [min]
X39031MS_8_01_12298.d: UV Chromatogram, 214-550 nm
X39031MS_8_01_12298.d: TIC +All MS

B

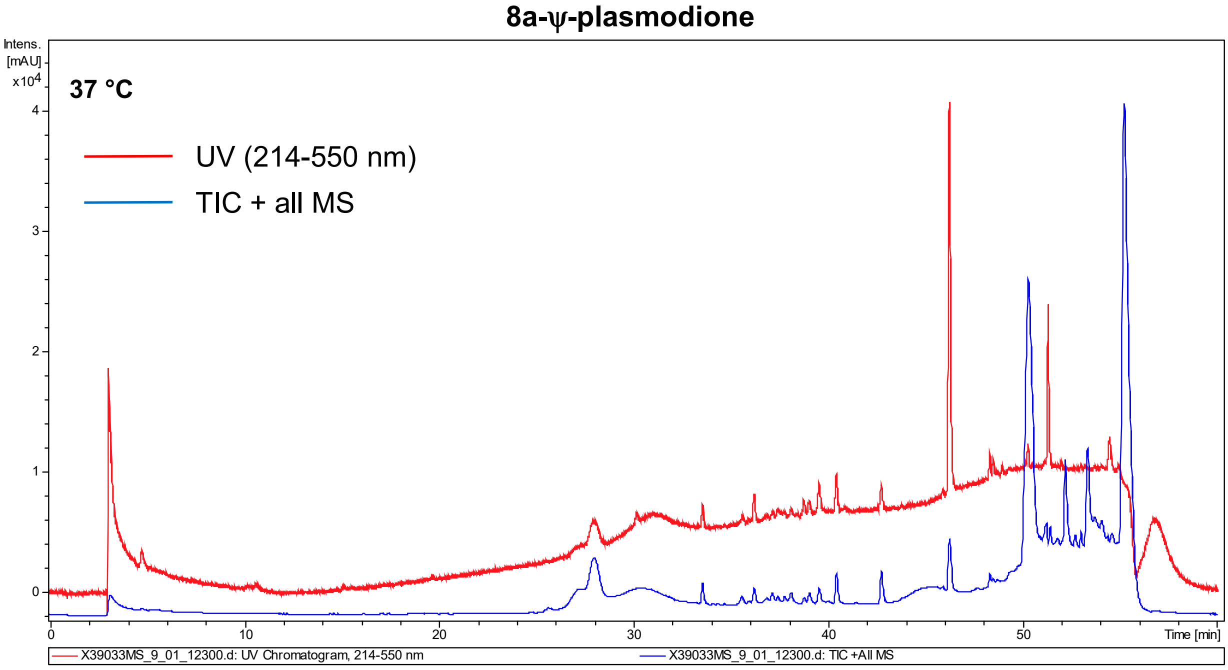
8a-ψ-plasmodione
Intens.
[mAU]
x10^4
37 °C
UV (214-550 nm)
TIC + all MS
4
3
2
1
0
0
10
20
30
40
50
Time [min]
X39033MS_9_01_12300.d: UV Chromatogram, 214-550 nm
X39033MS_9_01_12300.d: TIC +All MS

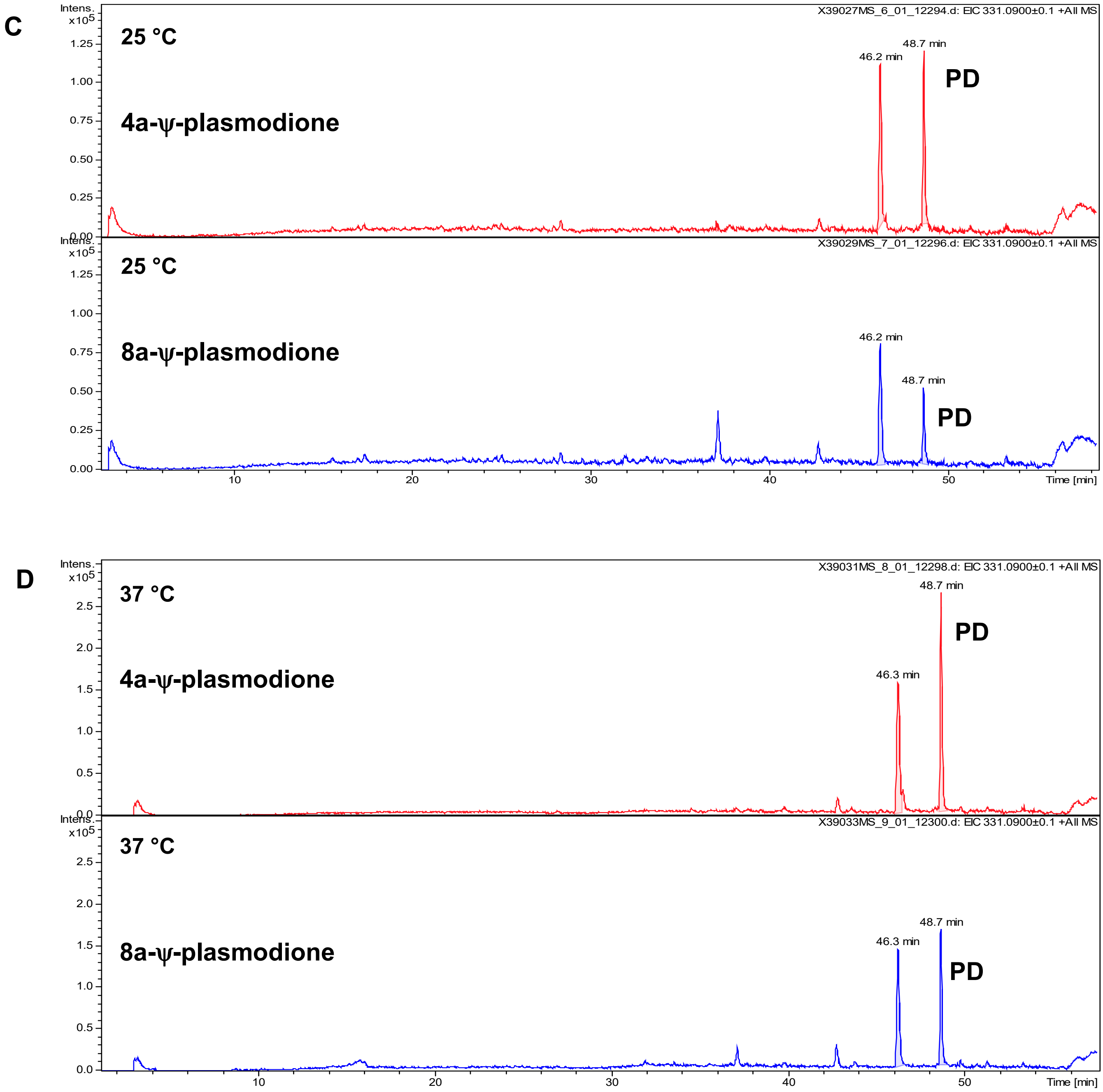

Figure S3. 280 nm UV-chromatogram overlaid with extracted Total Ion Chromatogram (TIC) obtained by LC-MS analysis of reaction mixtures maintained at 37 °C involving 4a-ψ-plasmodione **1** (Panel A) or 8a-ψ-plasmodione **2** (panel B). Comparison of the extracted ion chromatograms (EICs) corresponding to the mass of PD (RT = 48.7 min) after the same LC-MS analysis of reaction mixtures at 25 °C (Panel C) and 37 °C (Panel D).

NMR spectra

^{1}H NMR spectra of 4a-ψ-plasmodione 1.

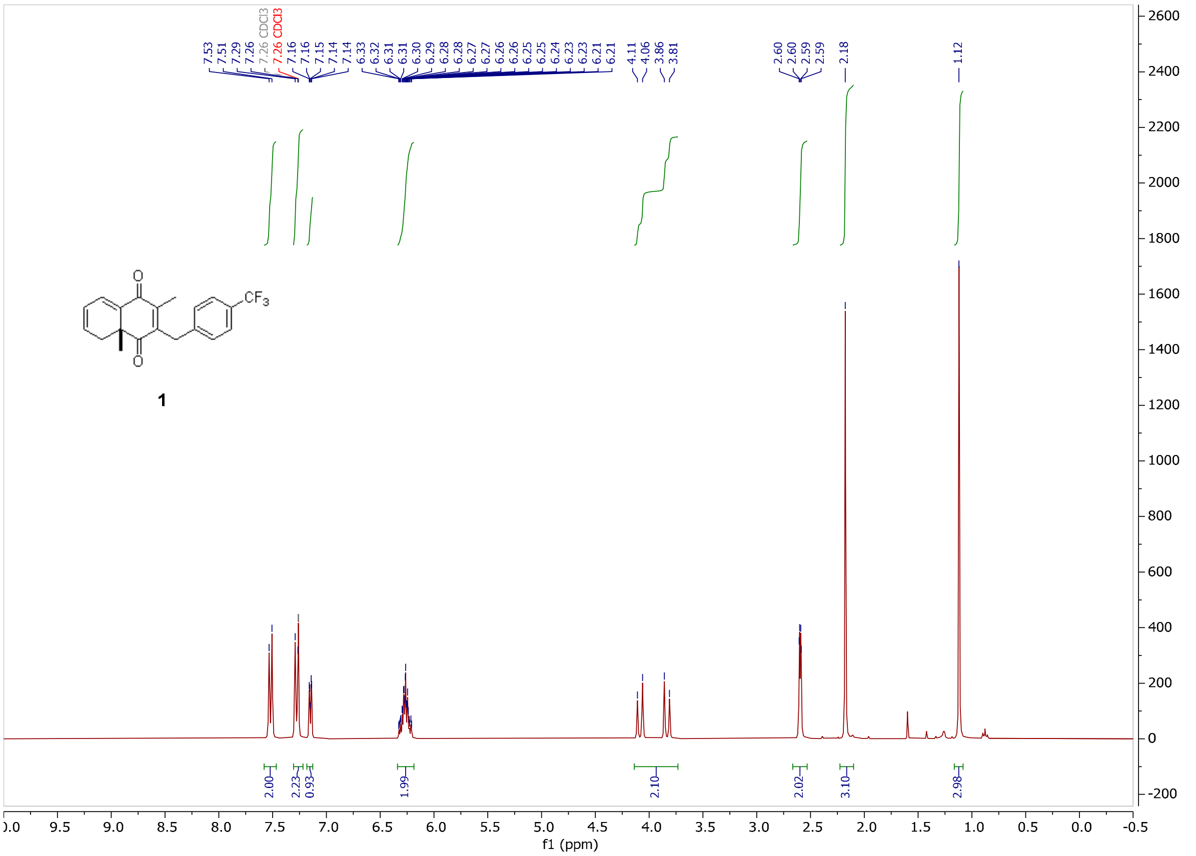

^{19}F {^{1}H} NMR spectra of 4a-ψ-plasmodione 1.

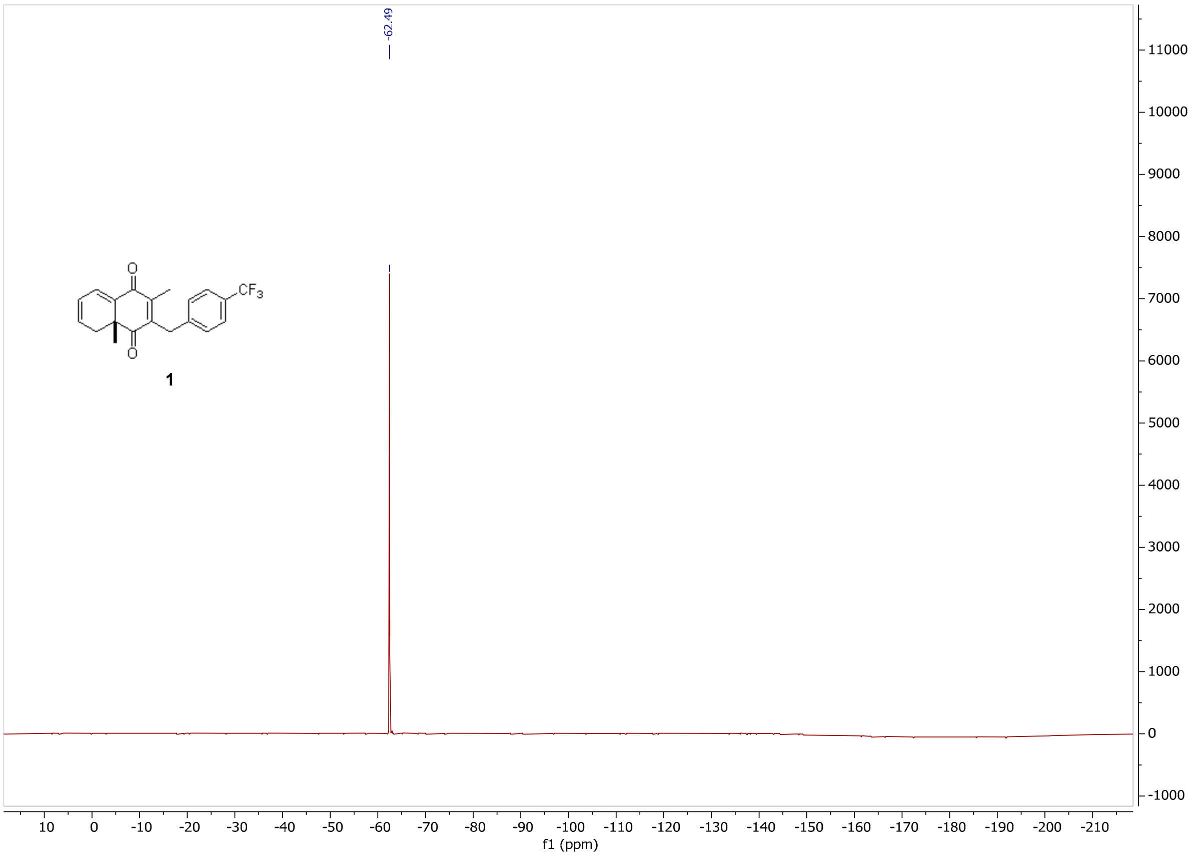

$^{13}C\{^1H\}$ NMR spectra of 4a-ψ-plasmodione 1.

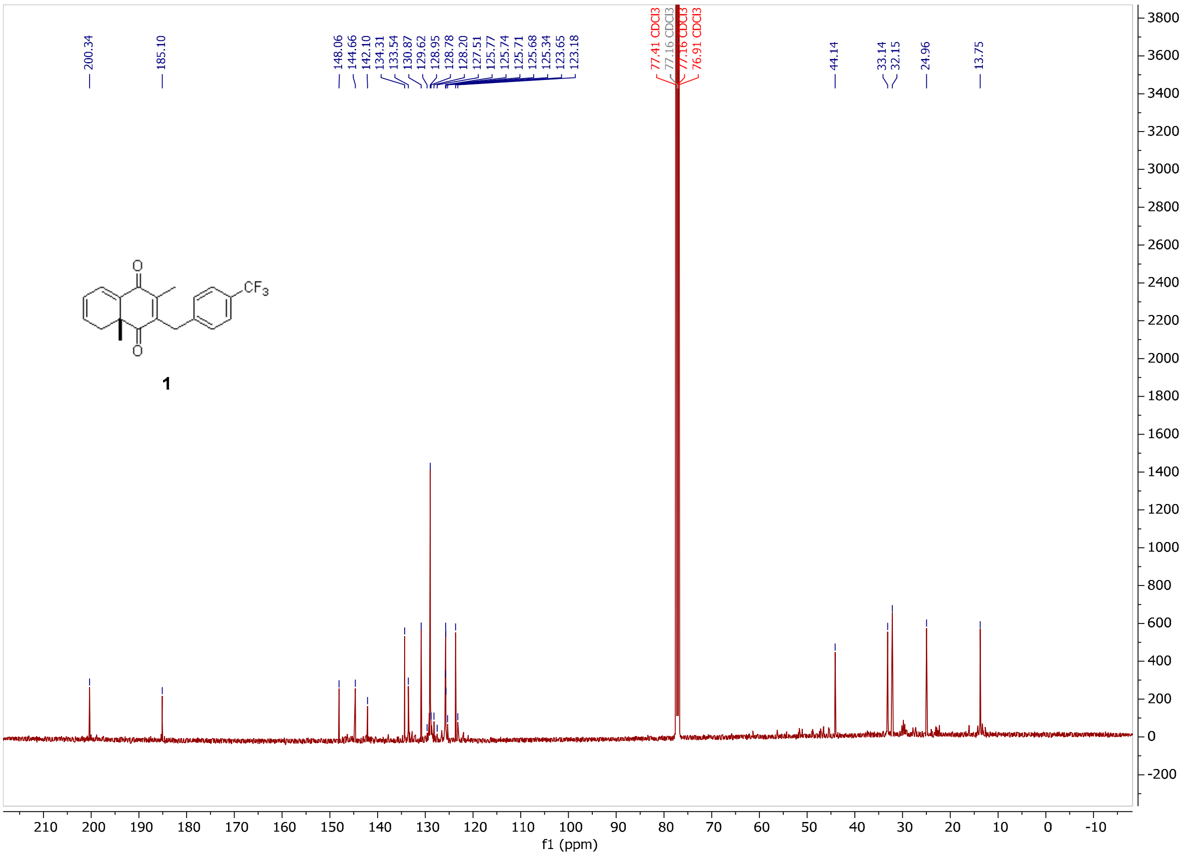

^1H NMR spectra of 8a-ψ-plasmodione 2.

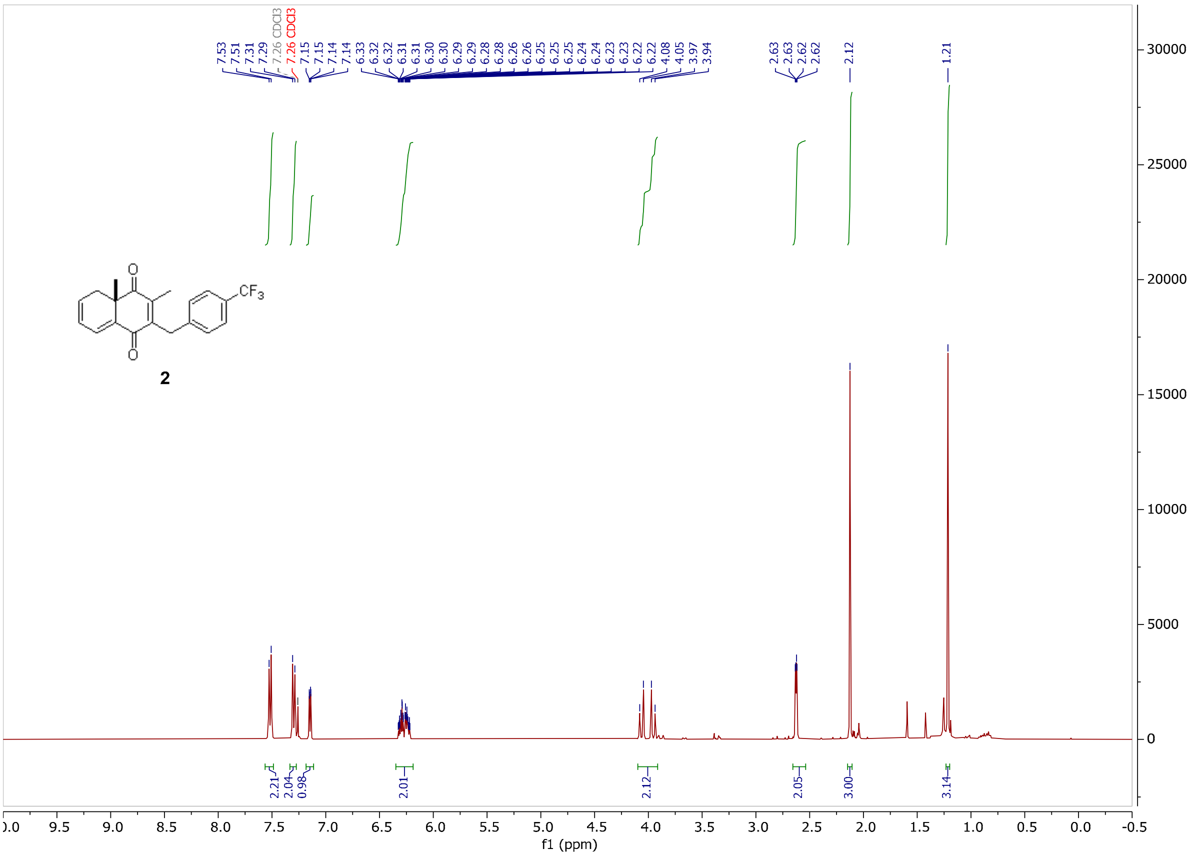

^{19}F {^{1}H} NMR spectra of 8a-ψ-plasmodione 2.

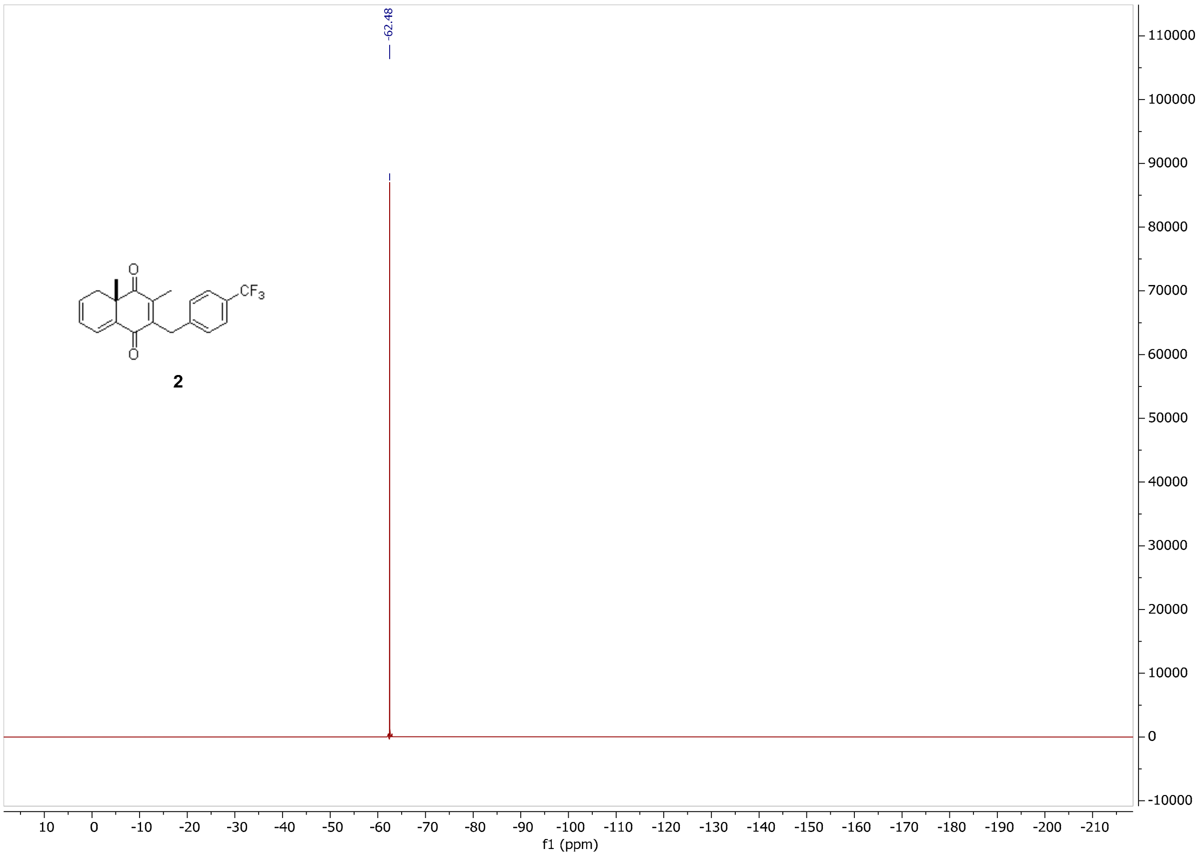

^{13}C {^{1}H} NMR spectra of 8a-ψ-plasmodione 2.

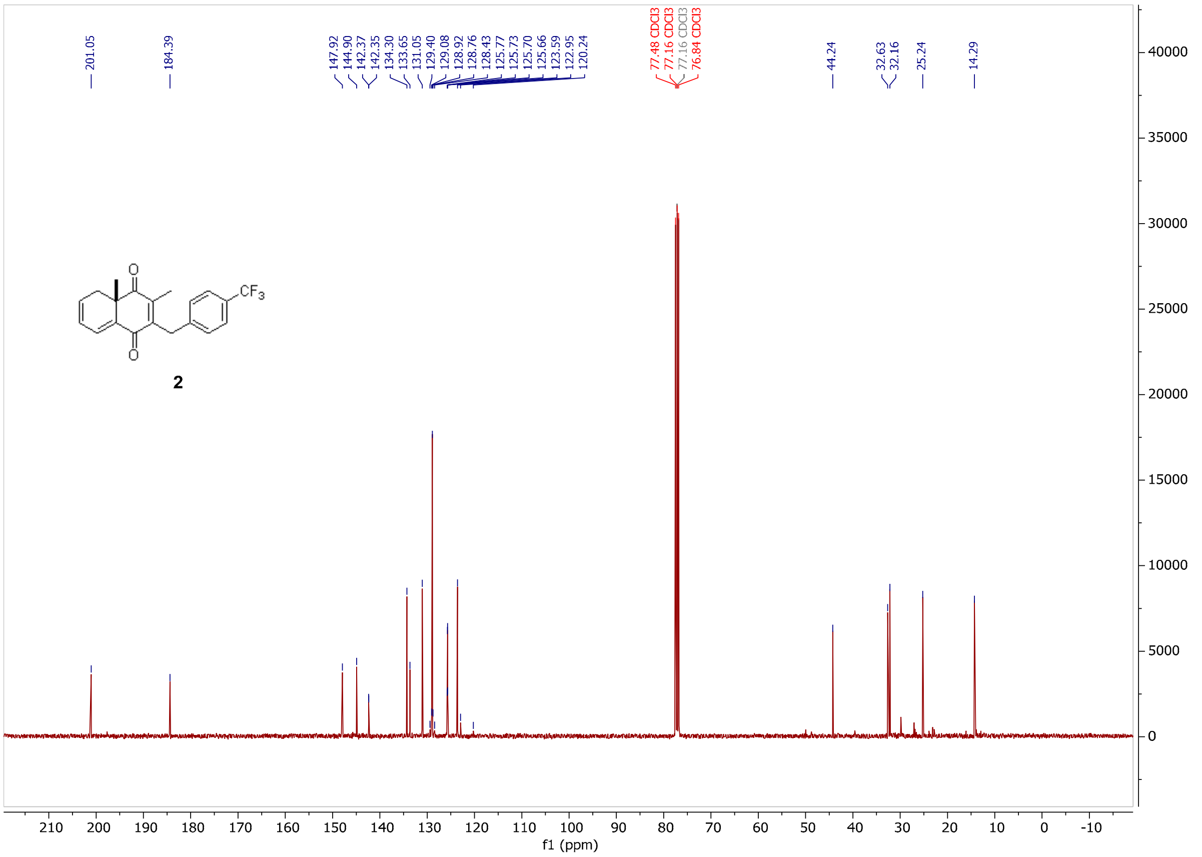

^{1}H NMR spectra of 4a-ψ-menadione 3.

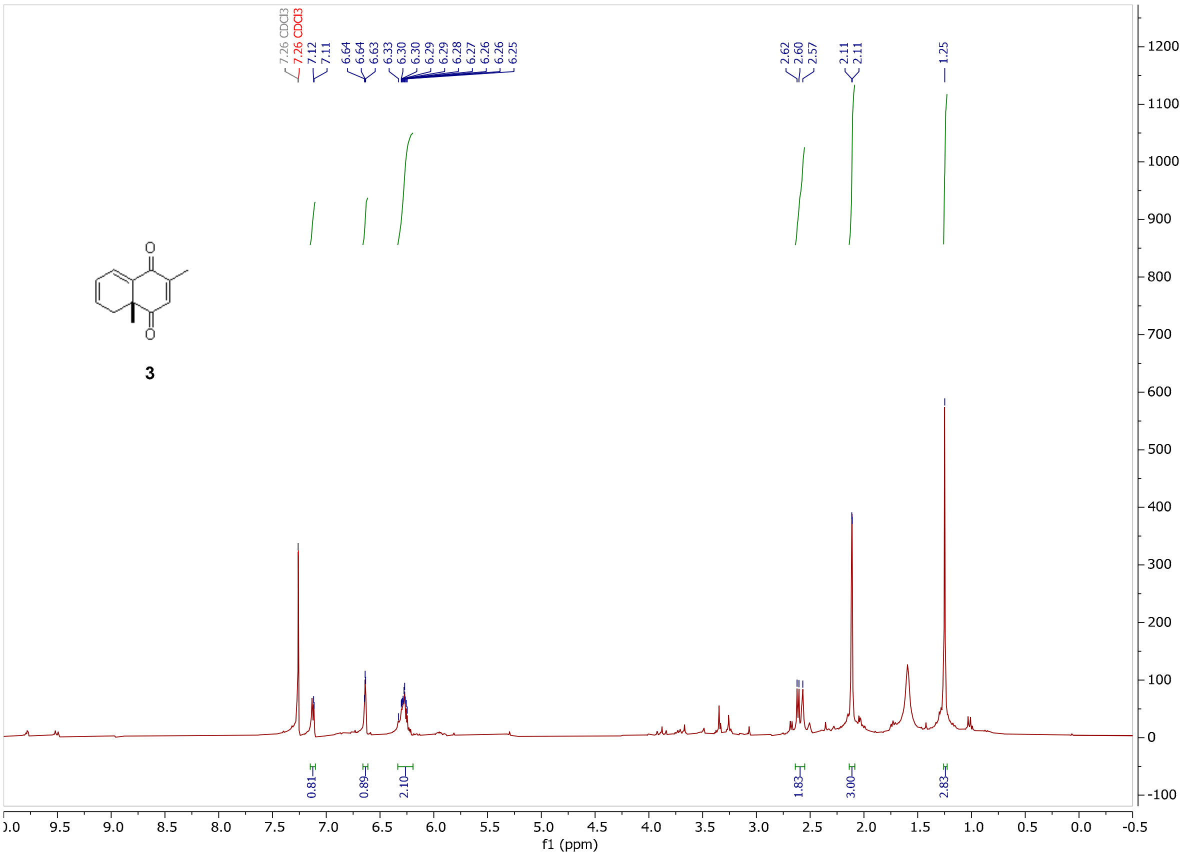

^{1}H NMR spectra of 8a-ψ-menadione 13.

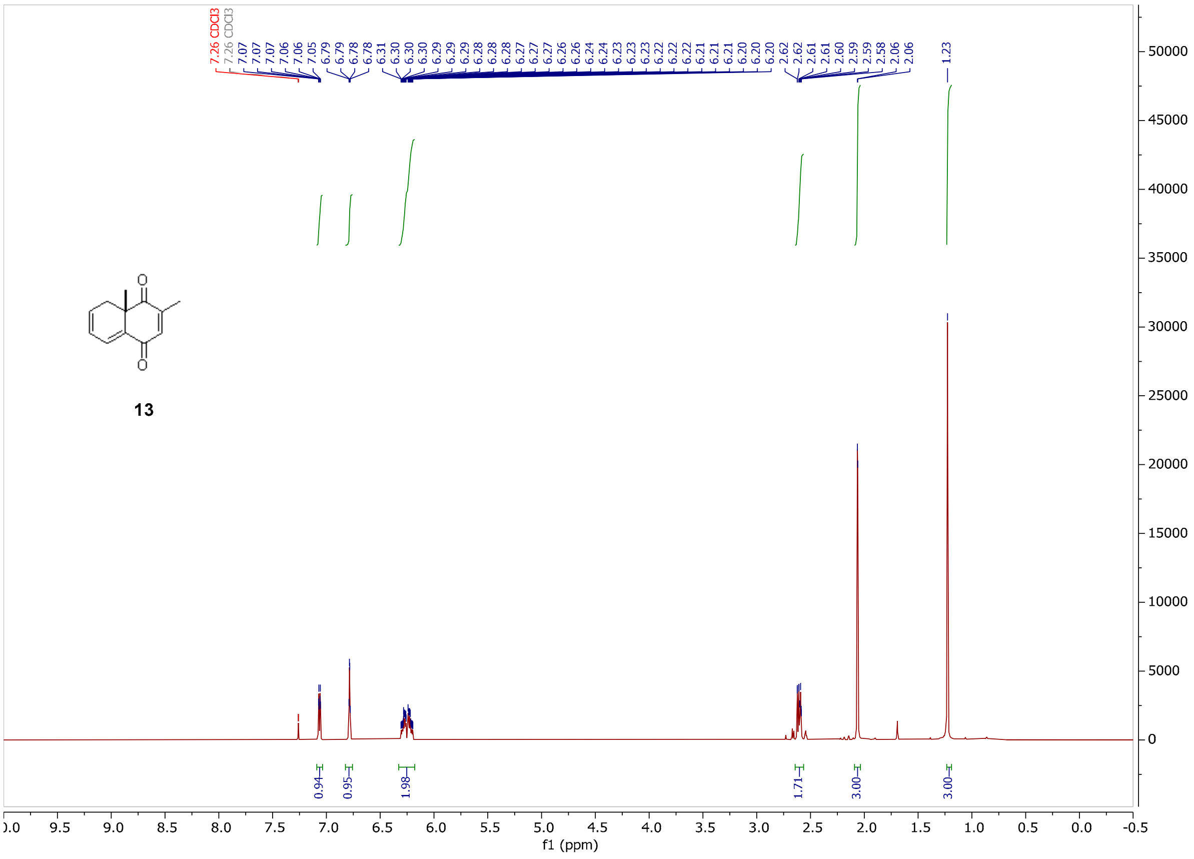

¹H NMR spectra of 16.

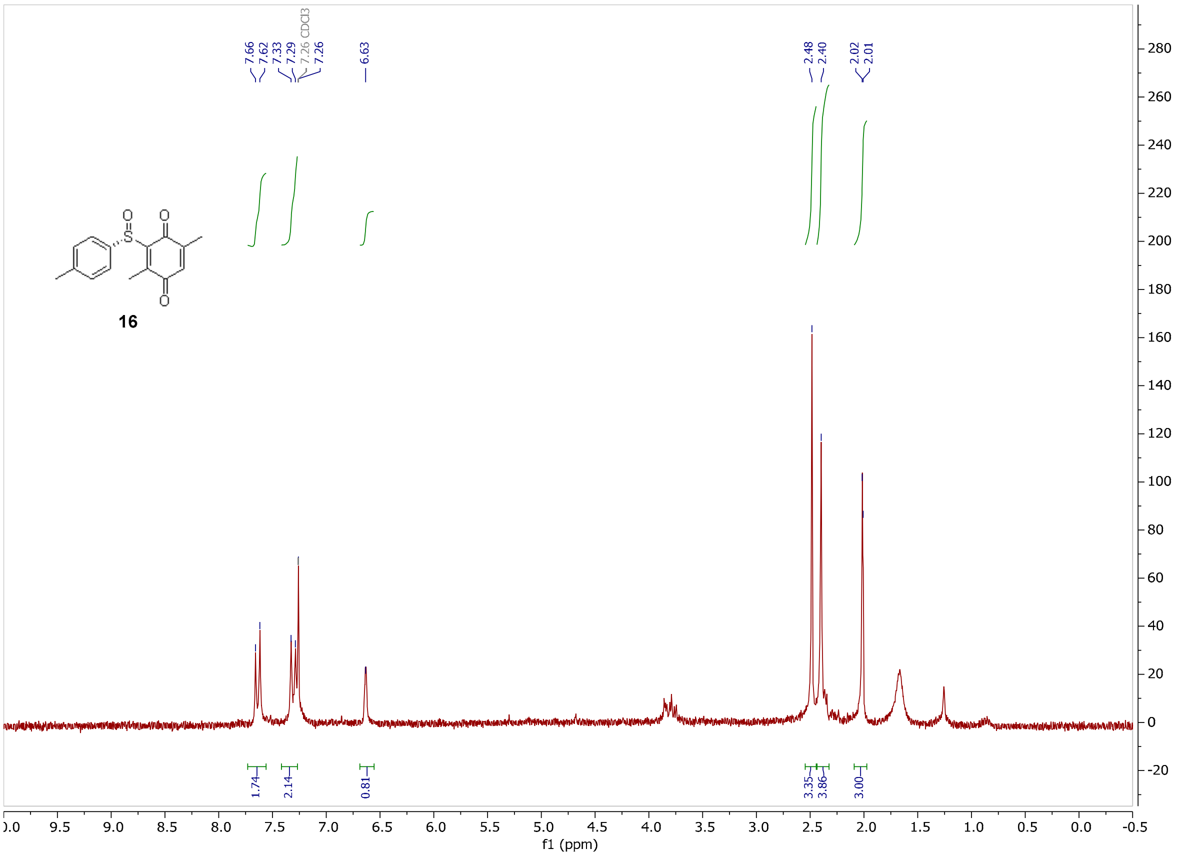

¹H NMR spectra of 17.

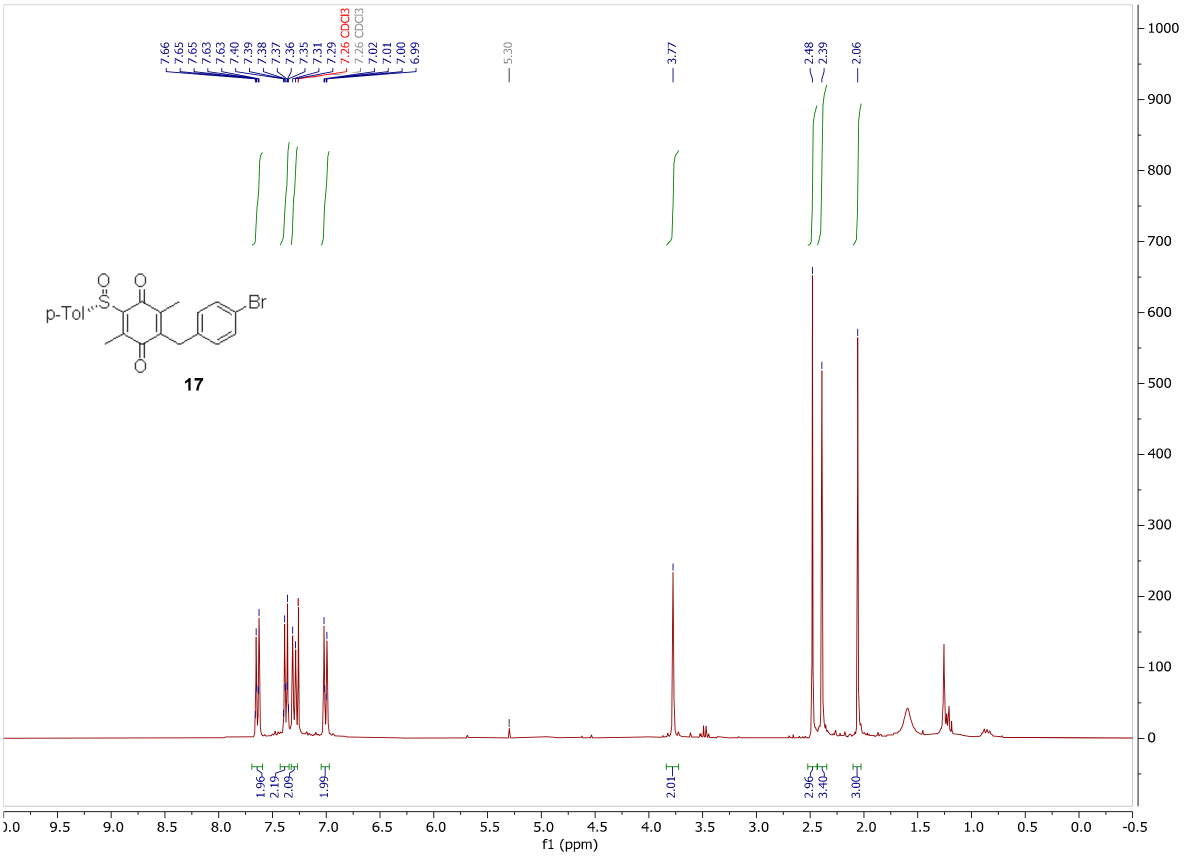

$^{13}C\{^1H\}$ NMR spectra of 17.

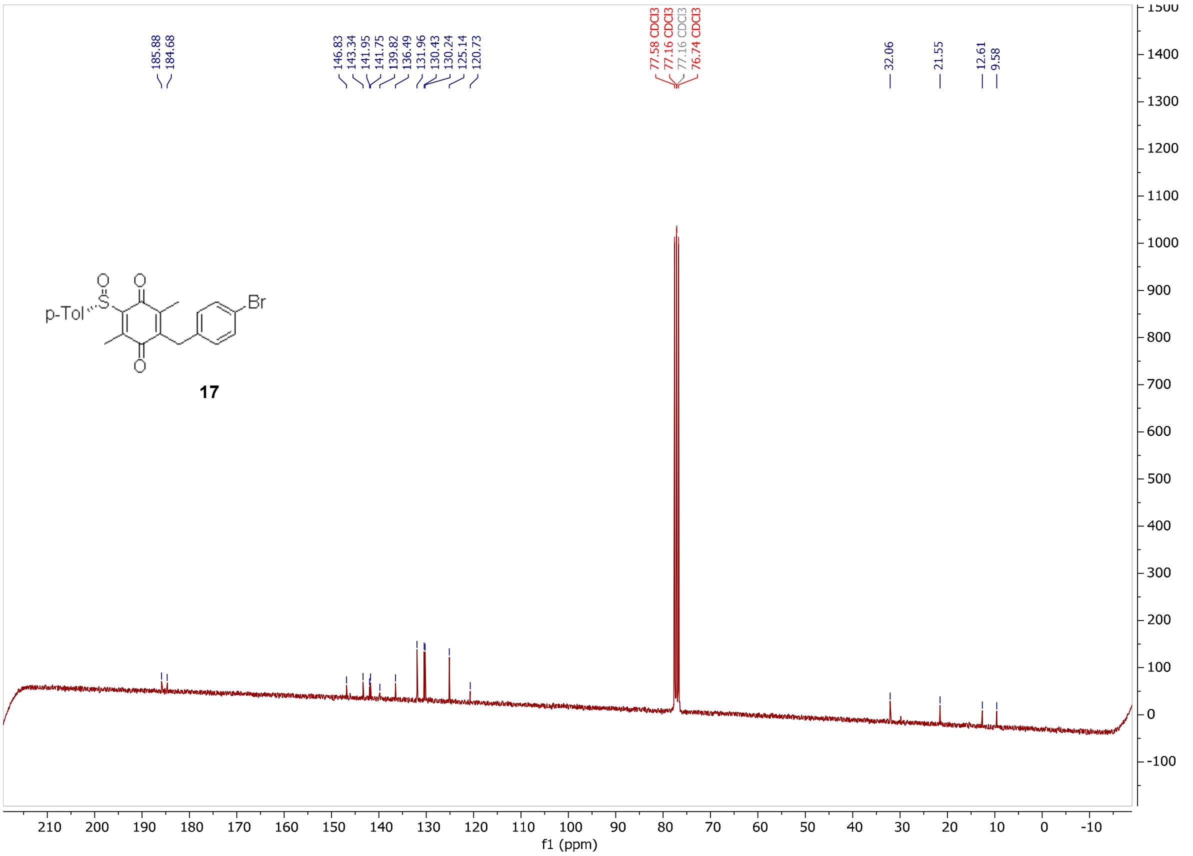

^1H NMR spectra of 19.

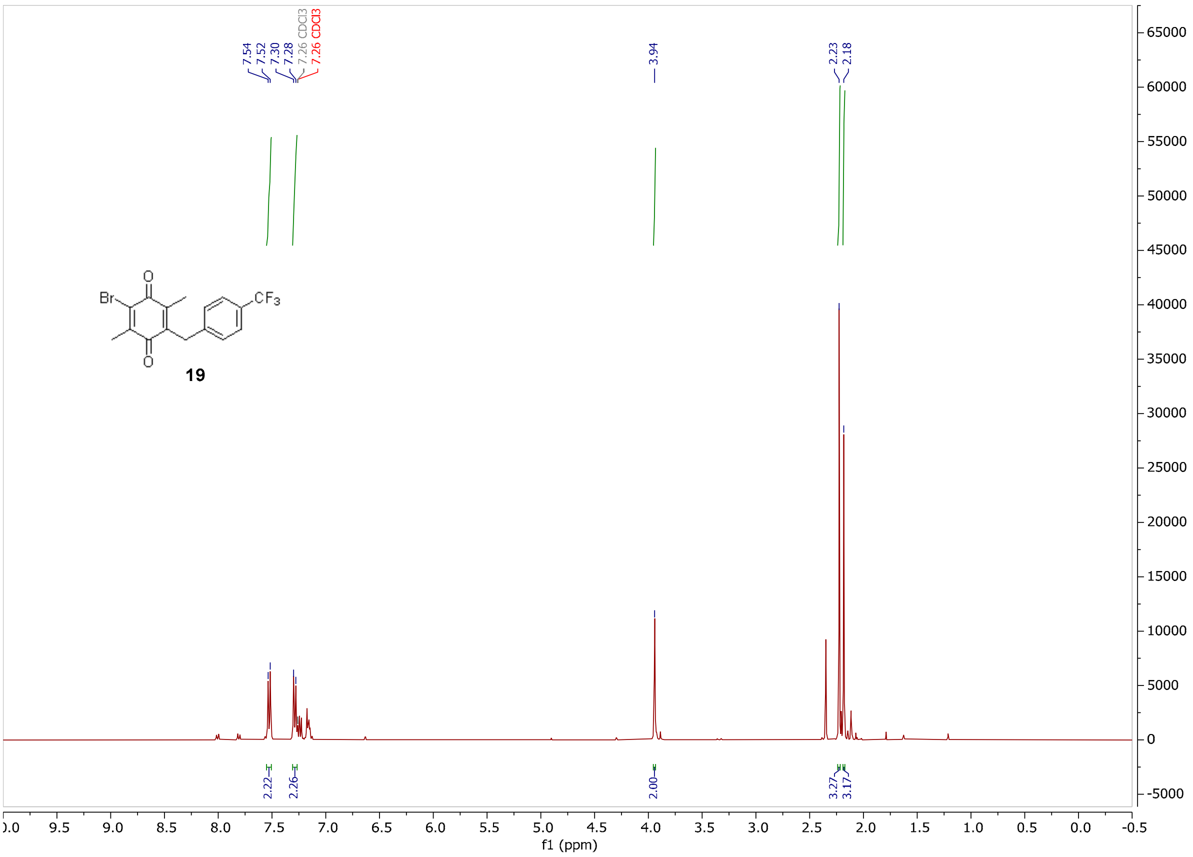

^{19}F {^{1}H} NMR spectra of 19.

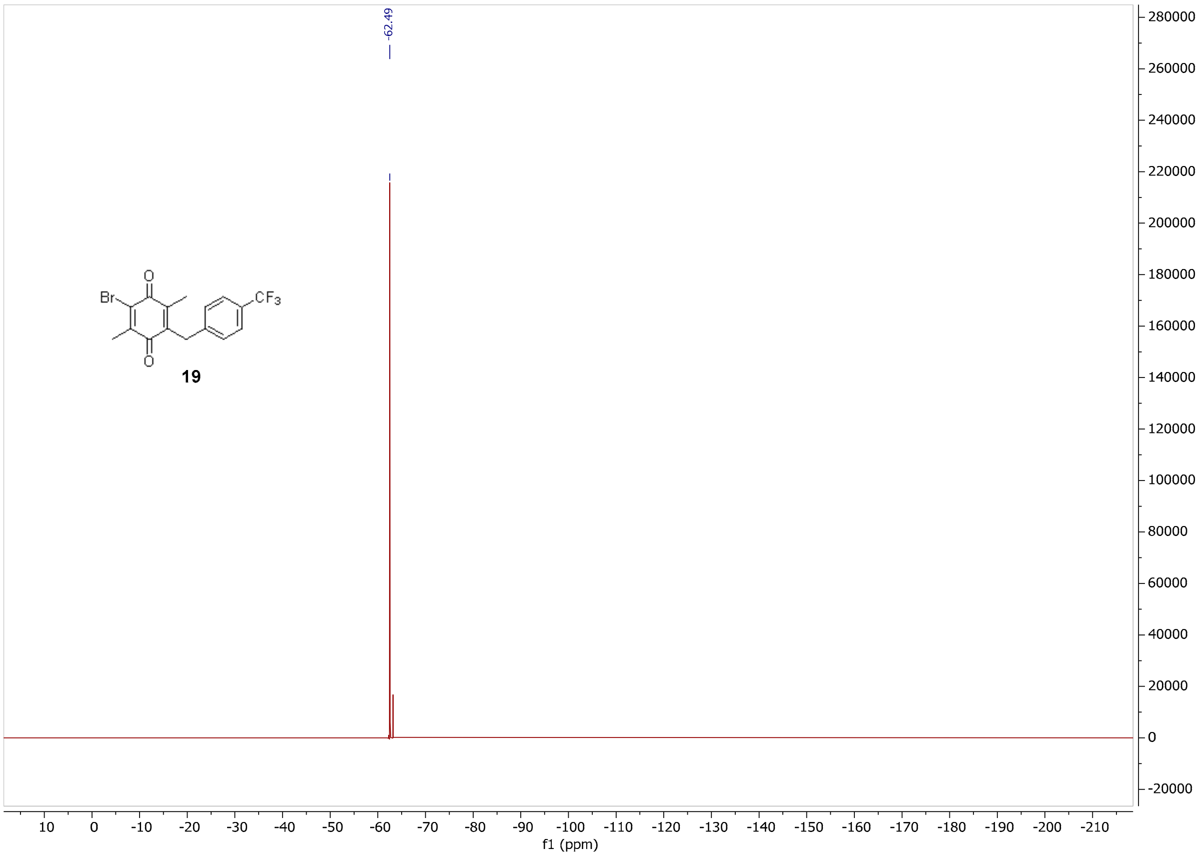

^{13}C {^{1}H} NMR spectra of 19.

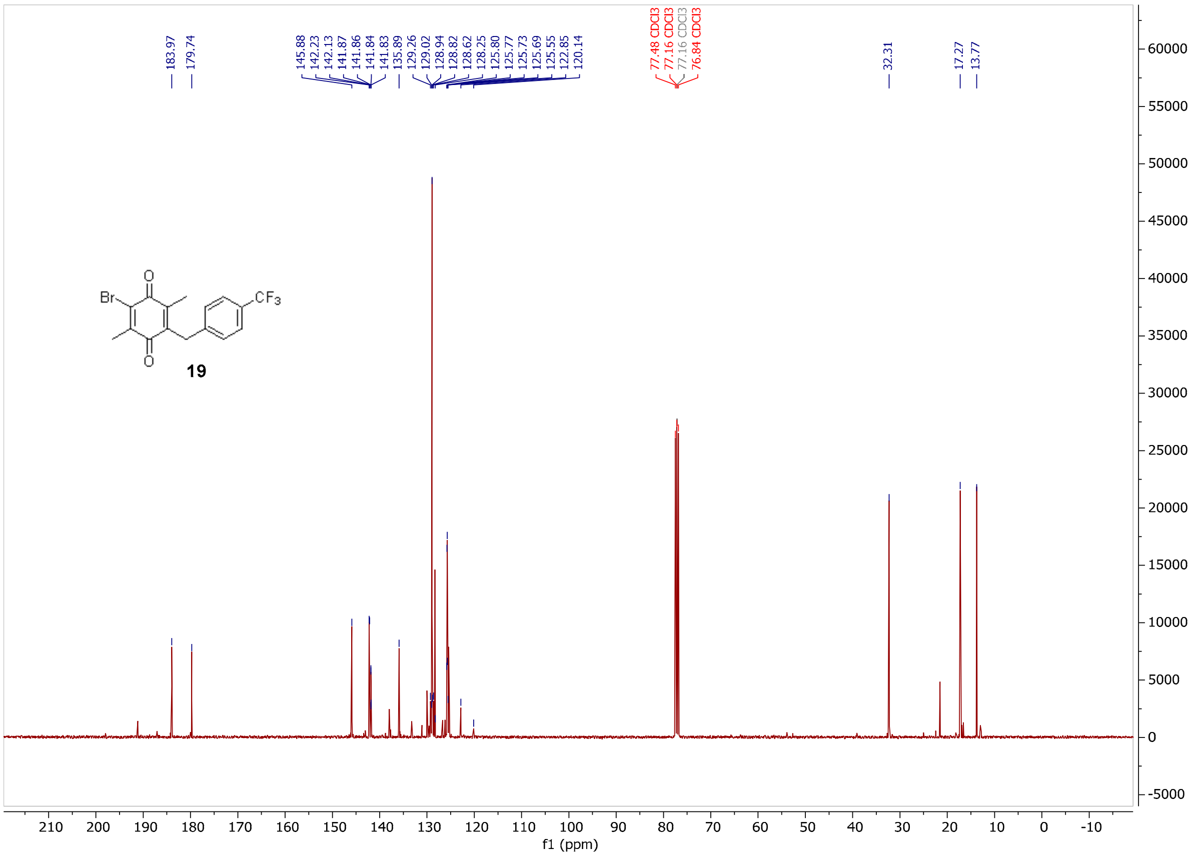

^{1}H NMR spectra of 20.

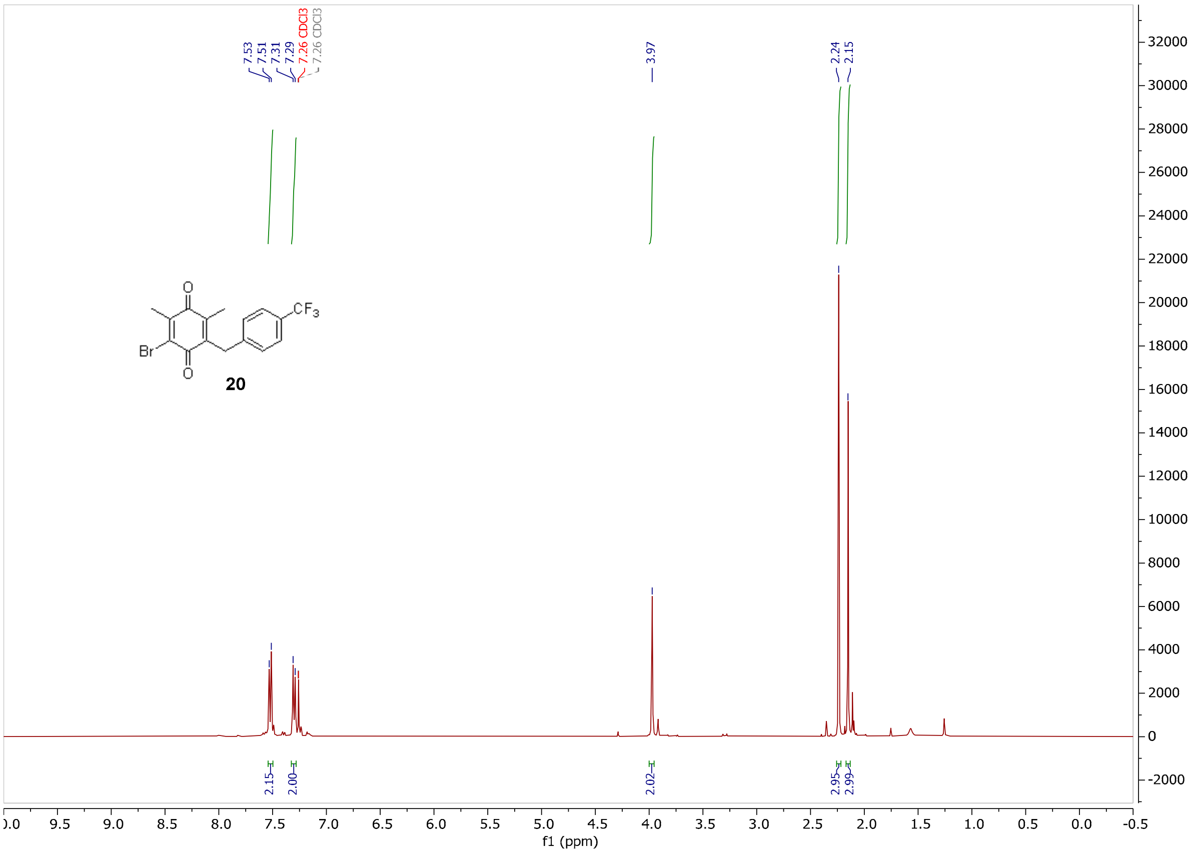

^{19}F {^{1}H} NMR spectra of 20.

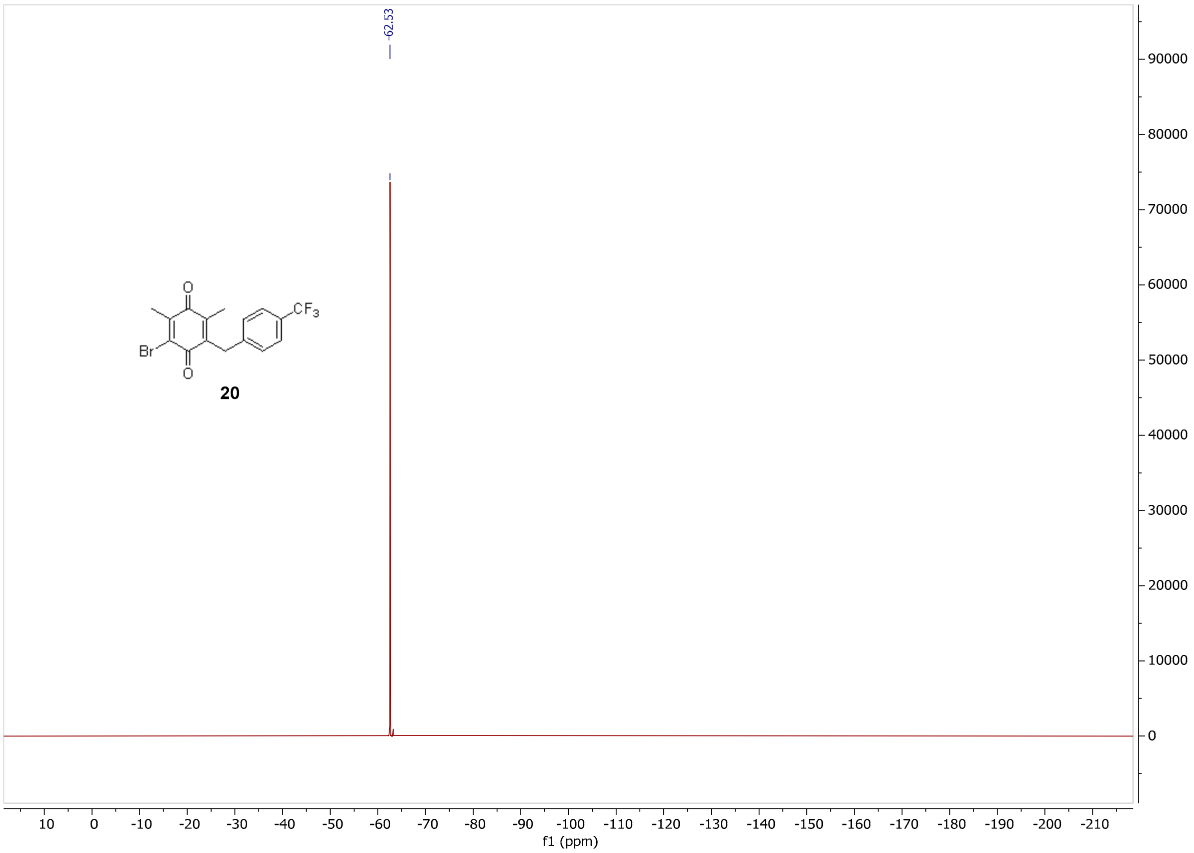

^{13}C {^{1}H} NMR spectra of 20.

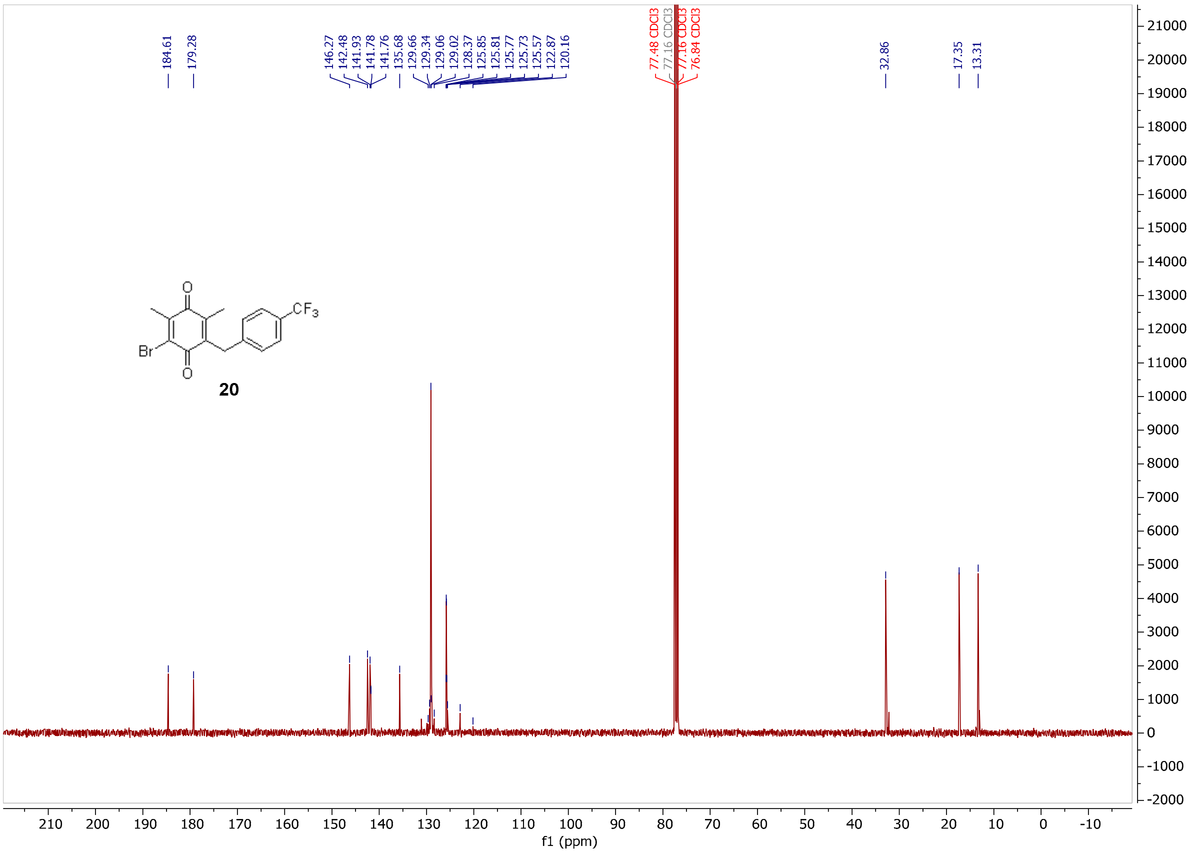

^{1}H NMR spectra of 21.

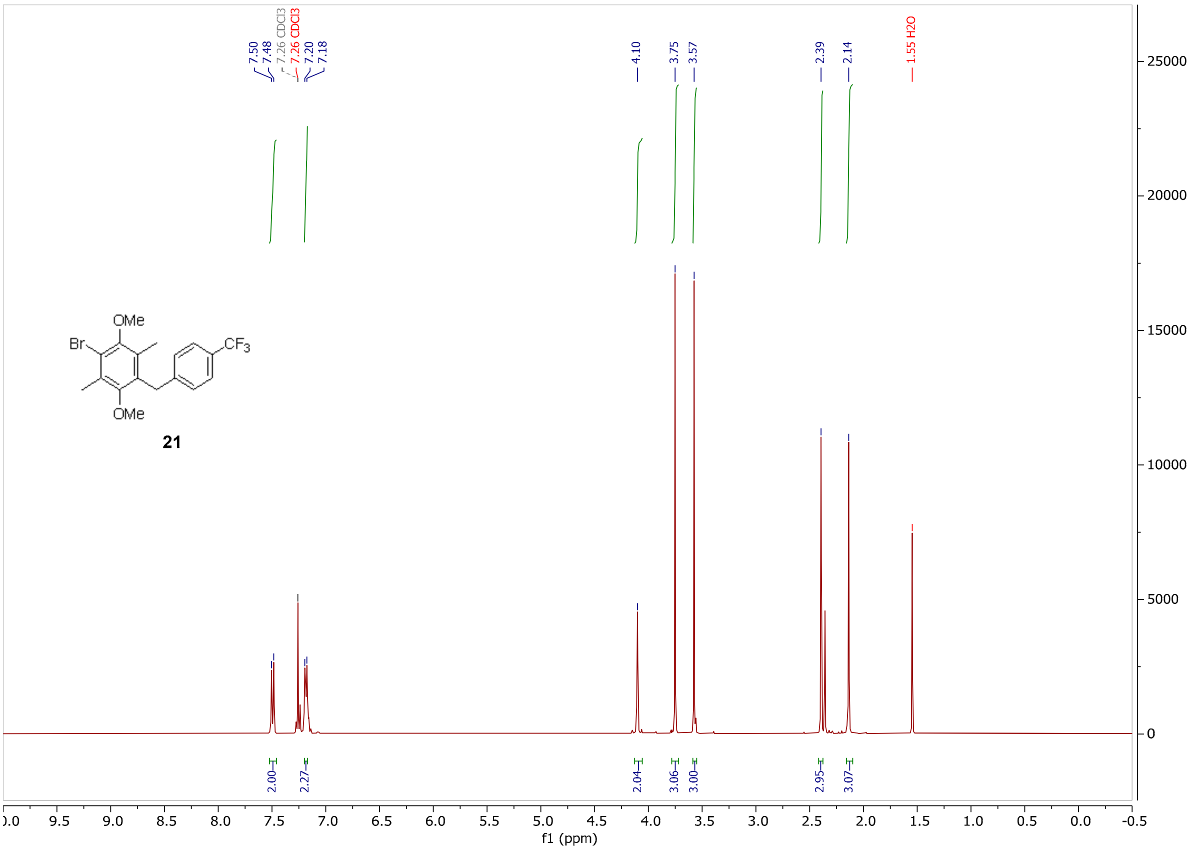

^{19}F {^{1}H} NMR spectra of 21.

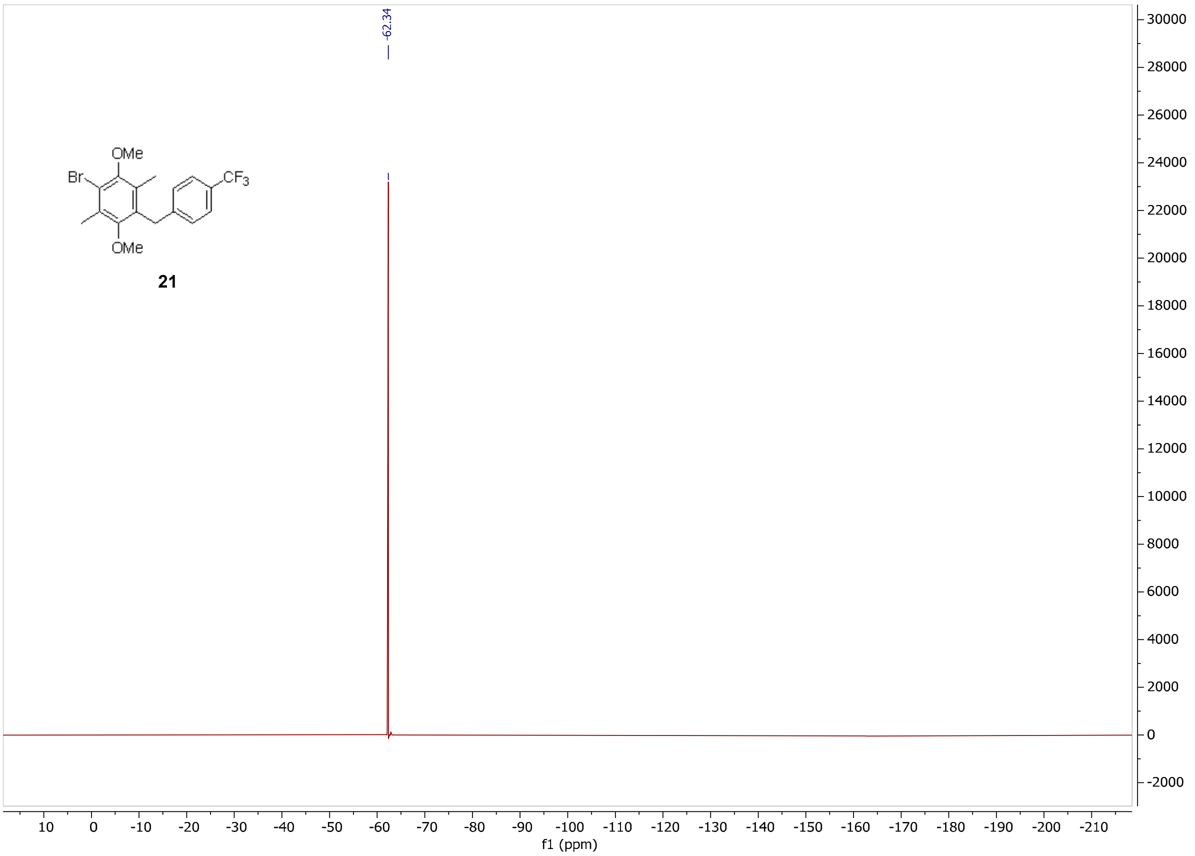

^{13}C {^{1}H} NMR spectra of 21.

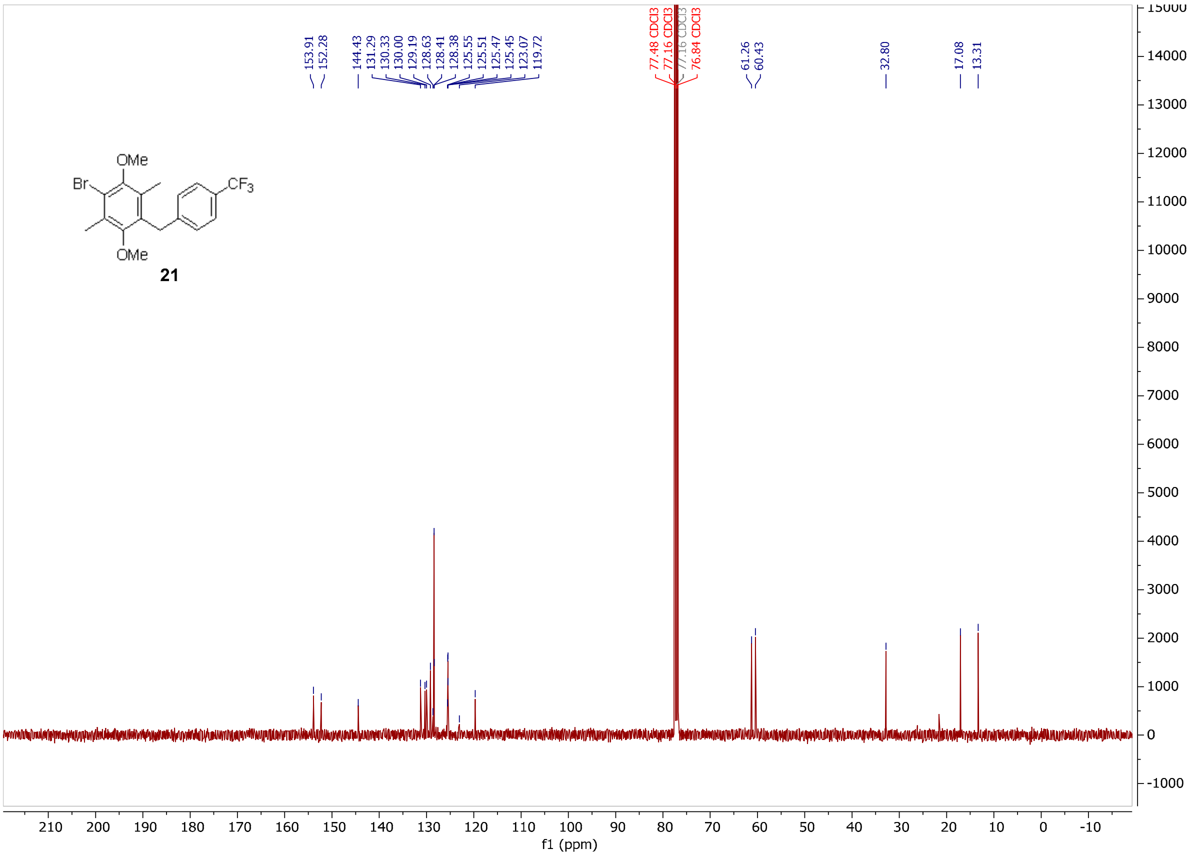

^{1}H NMR spectra of 22.

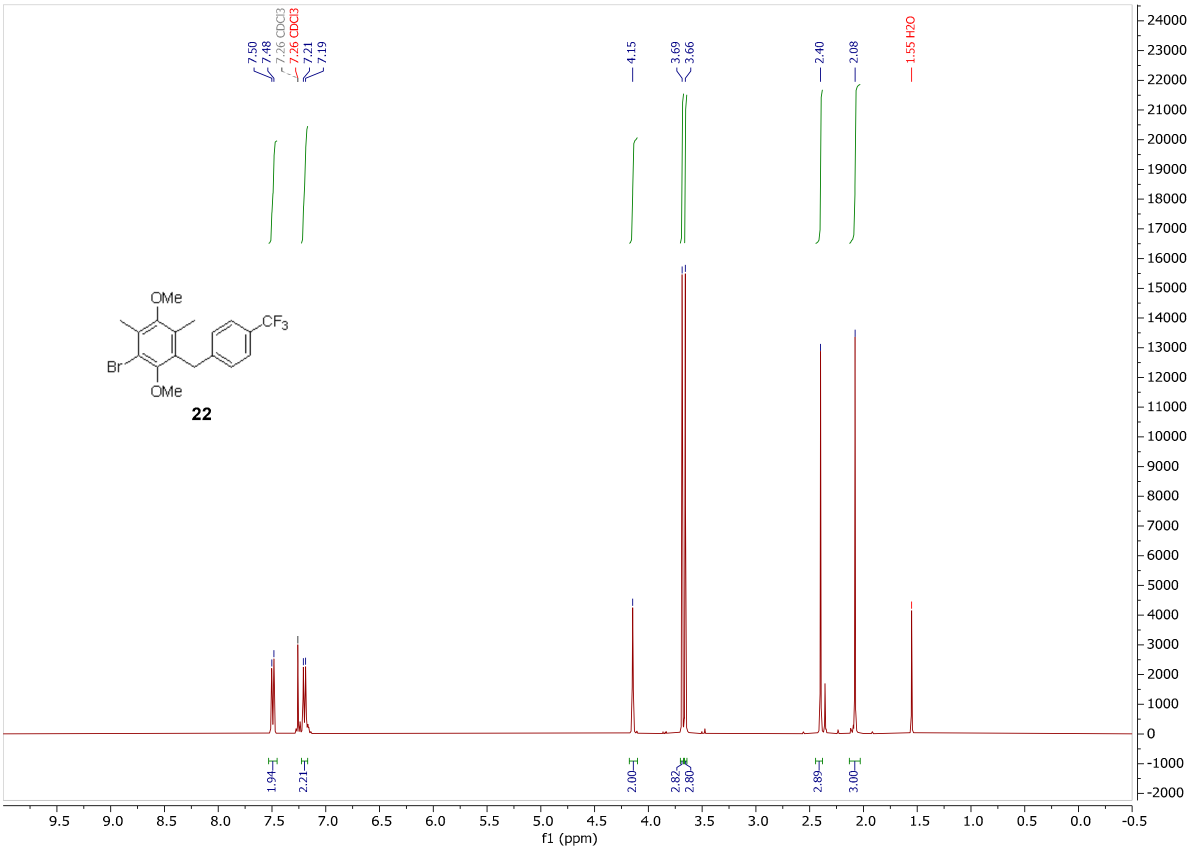

^{19}F {^{1}H} NMR spectra of 22.

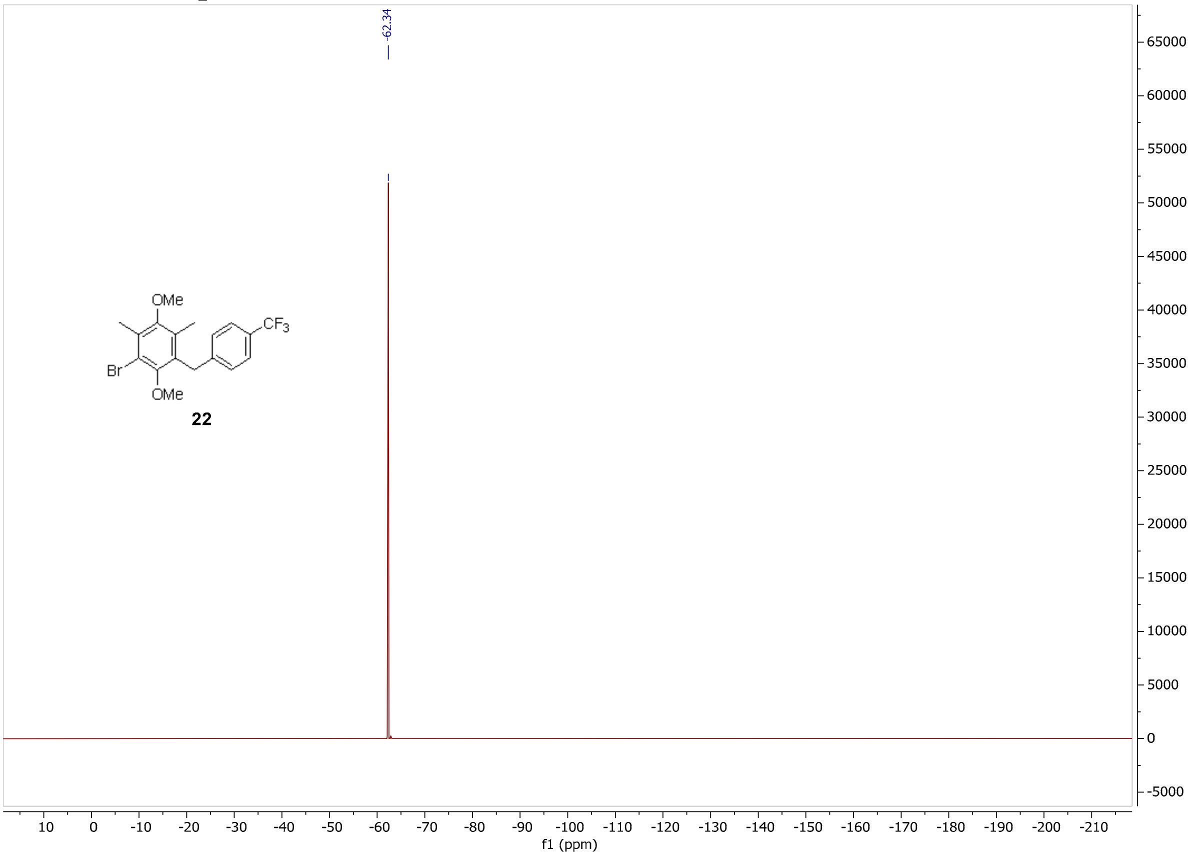

$^{13}C\{^{1}H\}$ NMR spectra of 22.

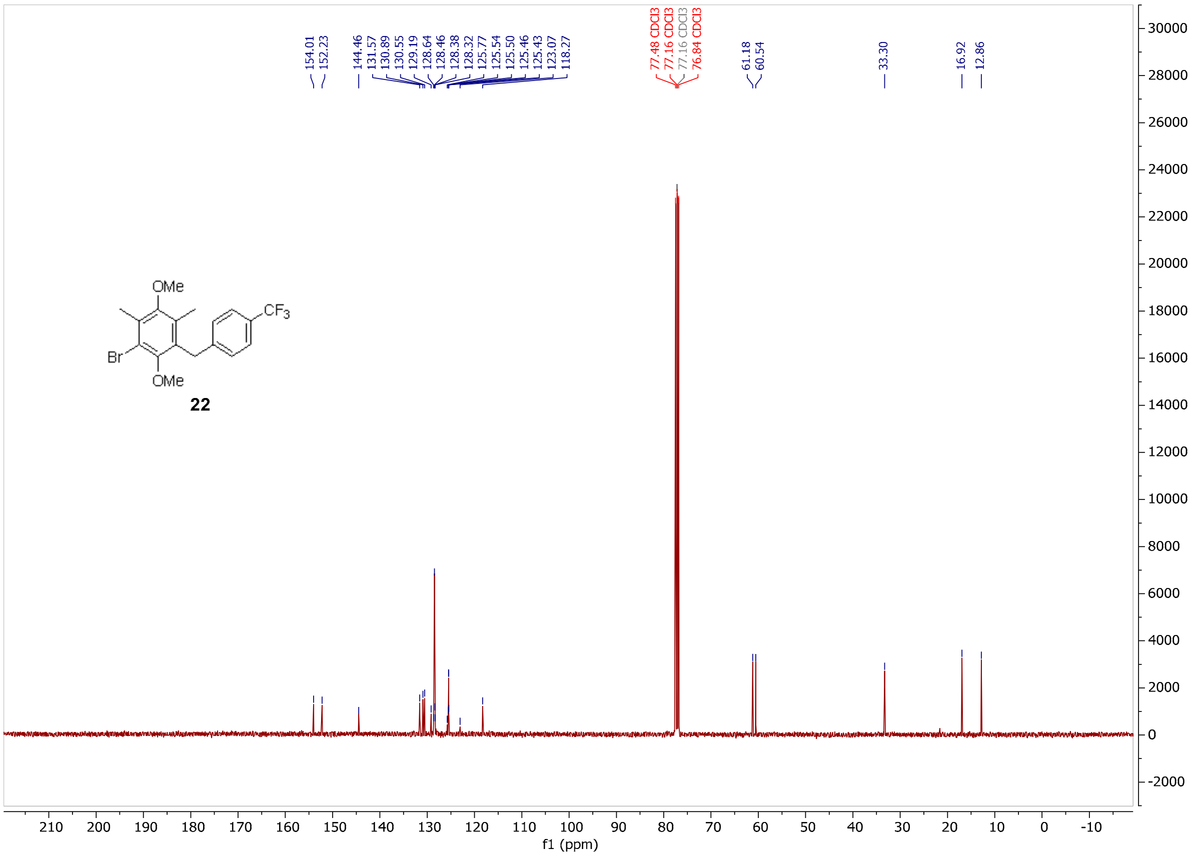

^{1}H NMR spectra of 24.

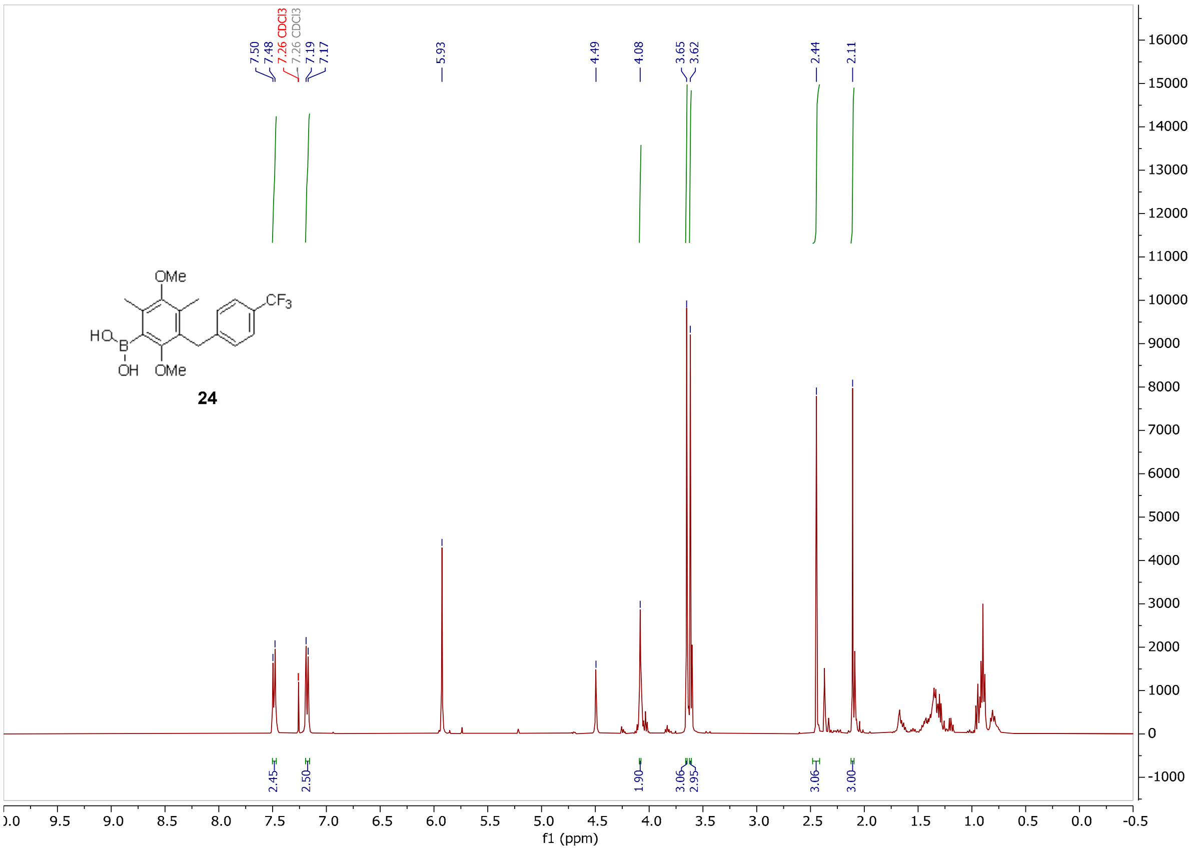

^{19}F {^{1}H} NMR spectra of 24.

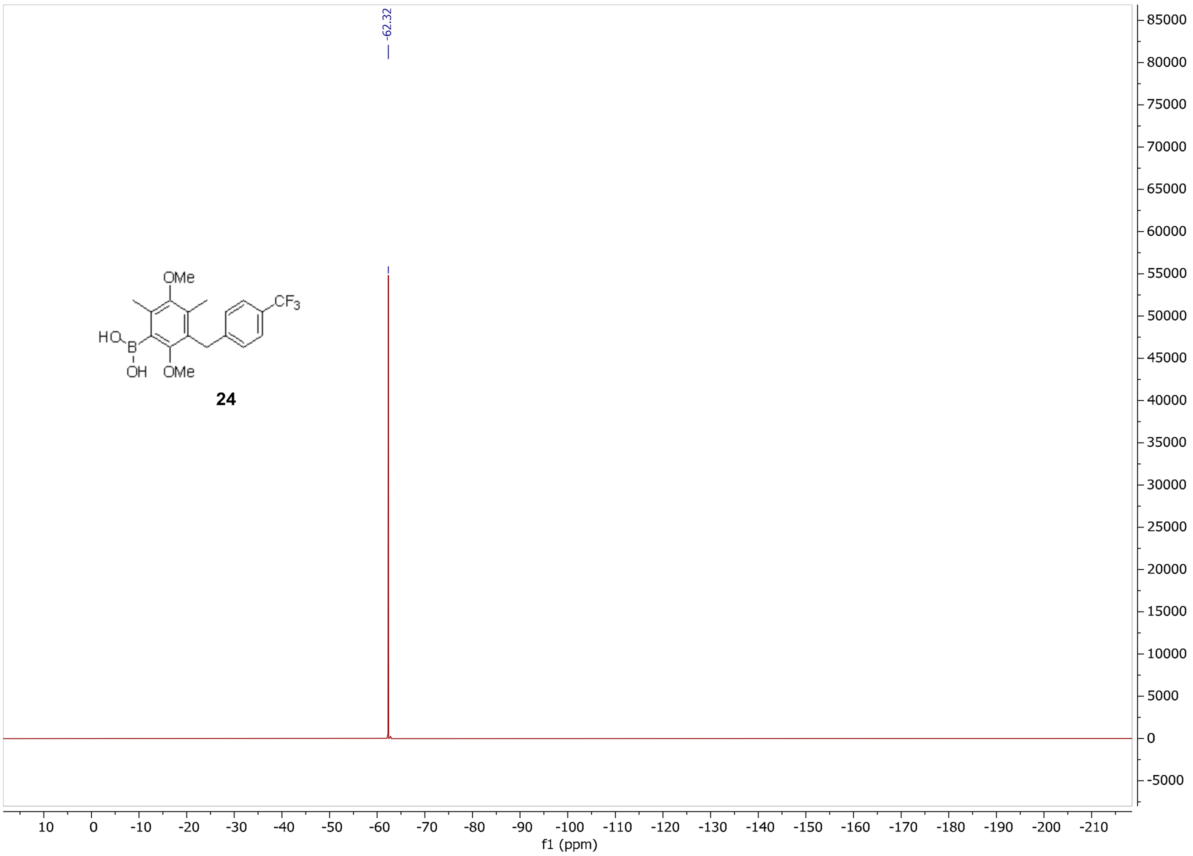

^{13}C {^{1}H} NMR spectra of 24.

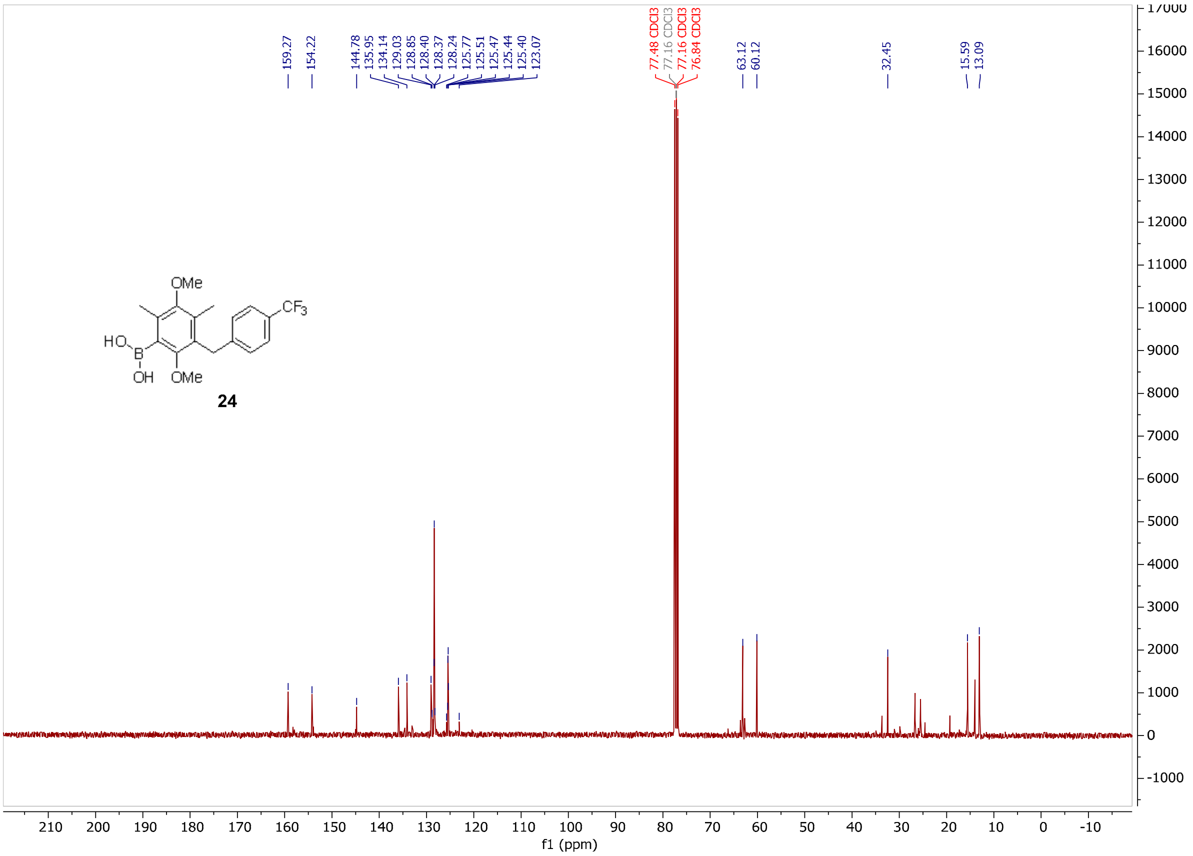

^{1}H NMR spectra of 25.

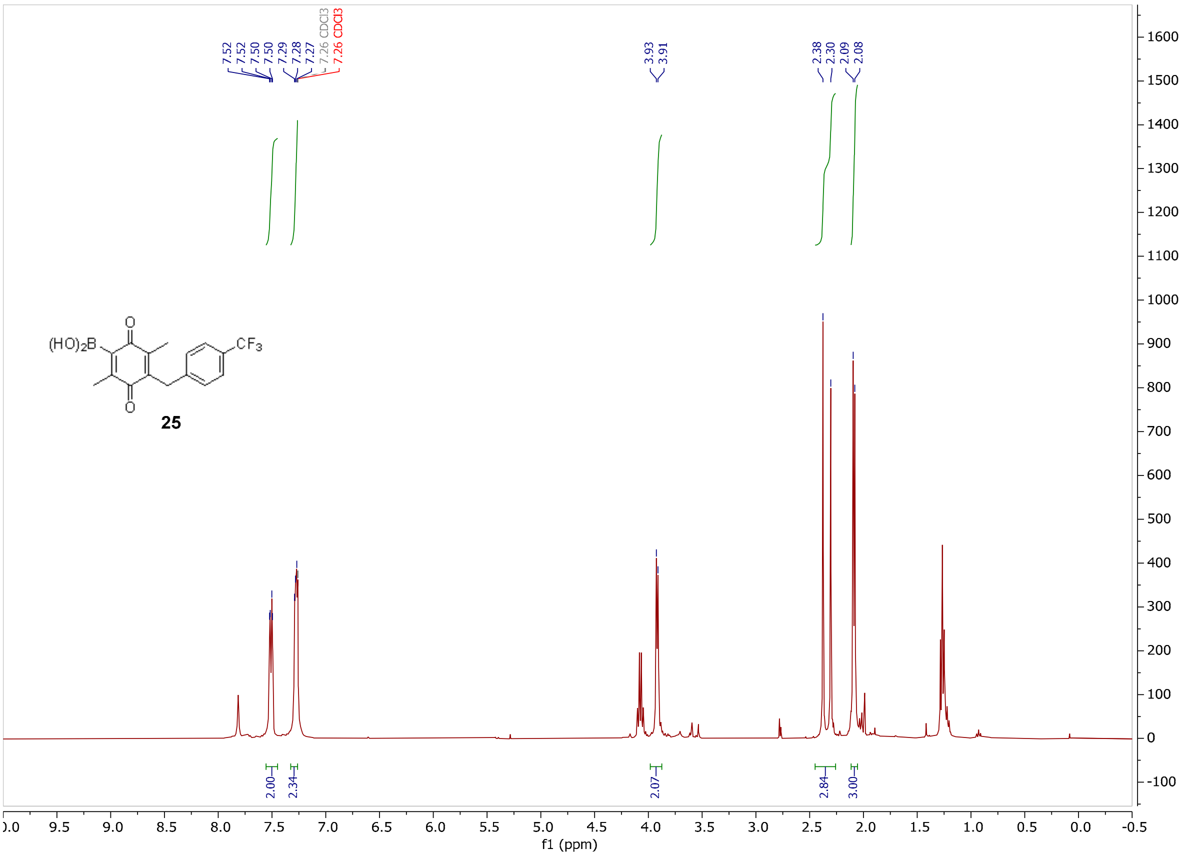

^{19}F {^{1}H} NMR spectra of 25

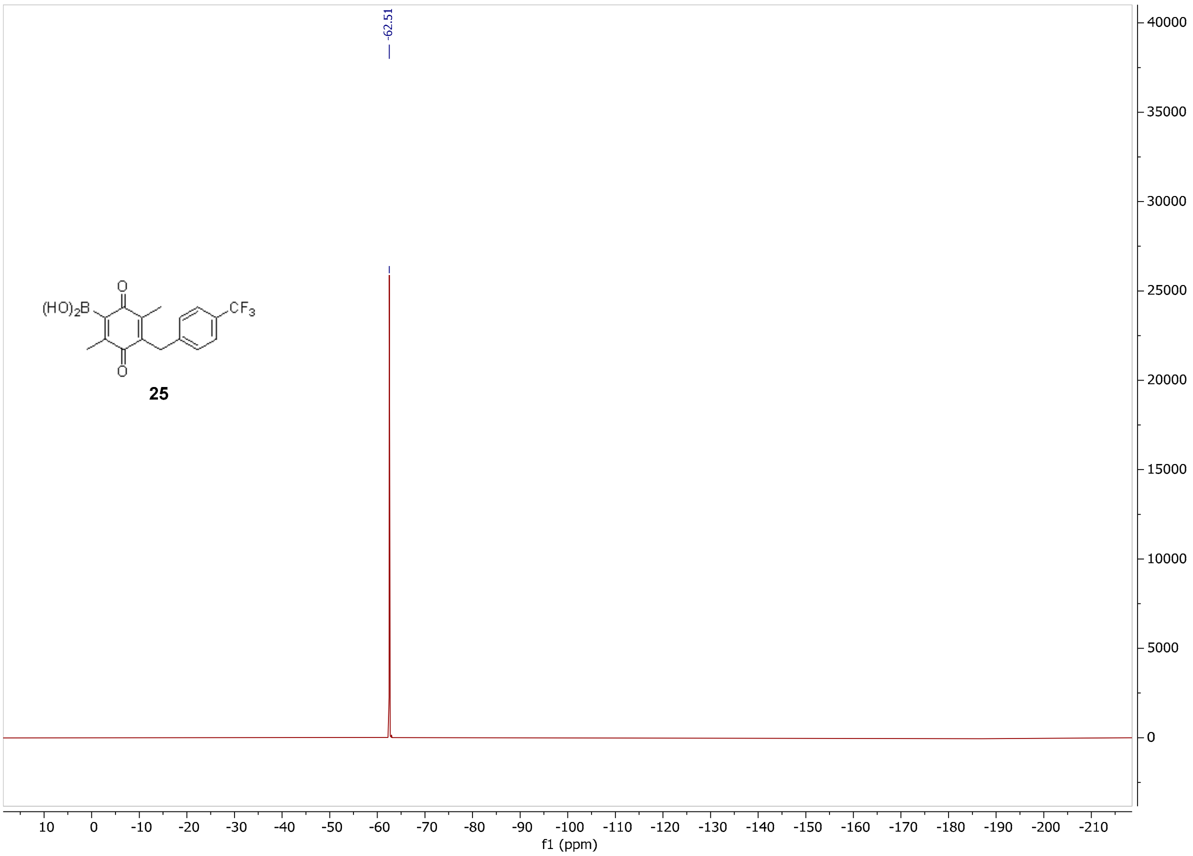

^{13}C {^{1}H} NMR spectra of 25.

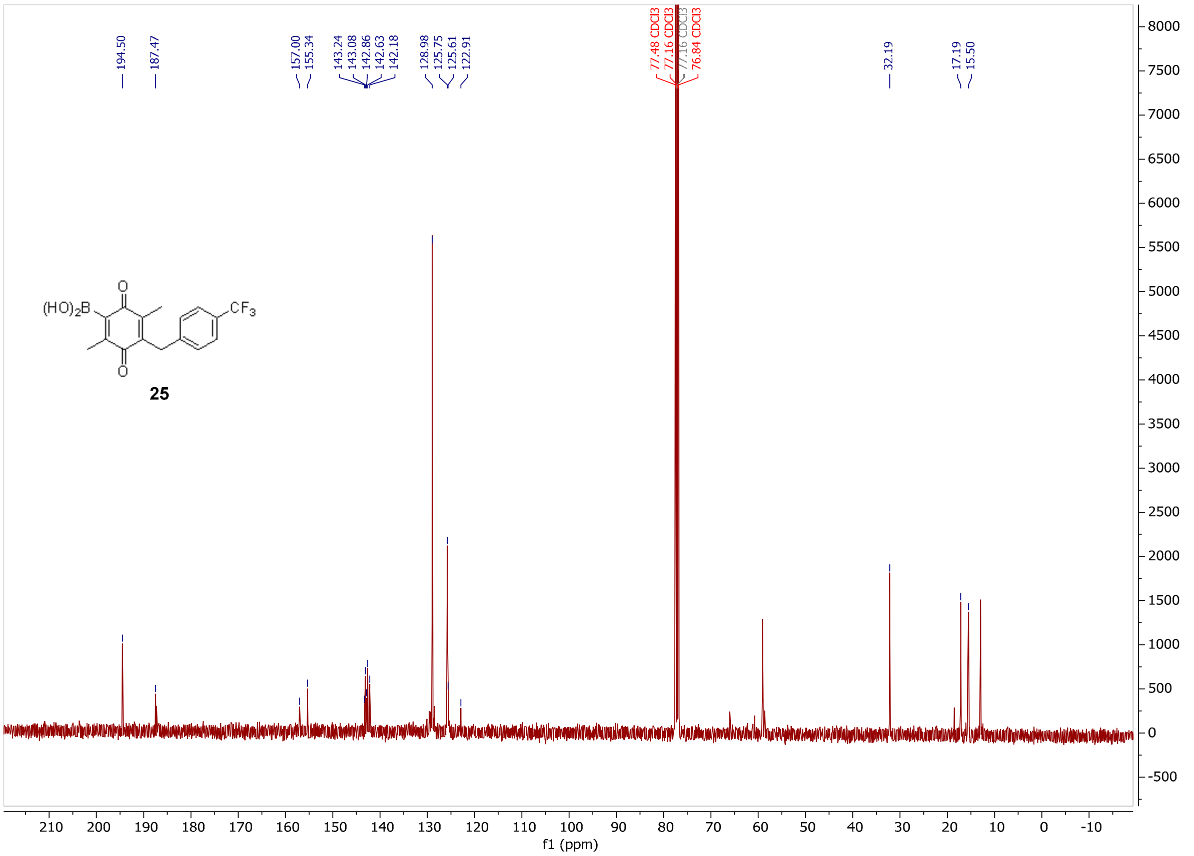

^{1}H NMR spectra of 27.

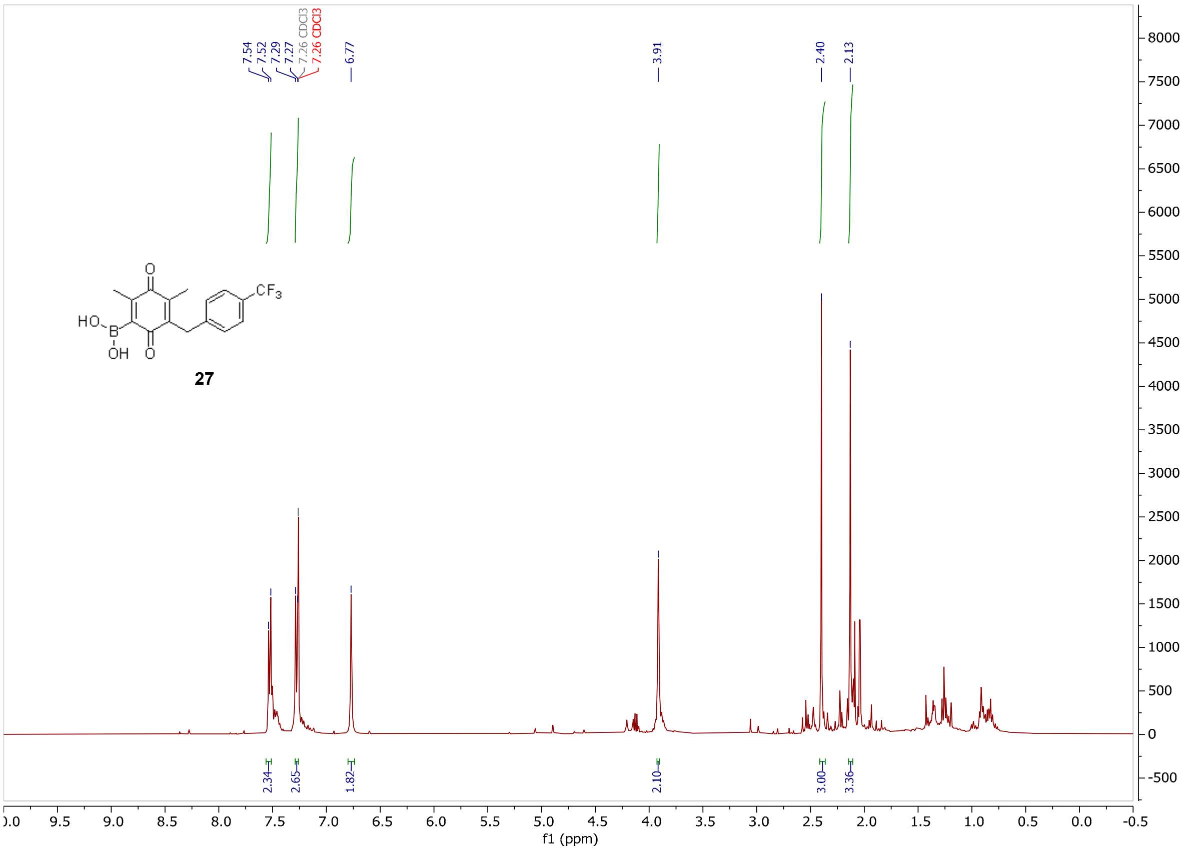

^{19}F {^{1}H} NMR spectra of 27.

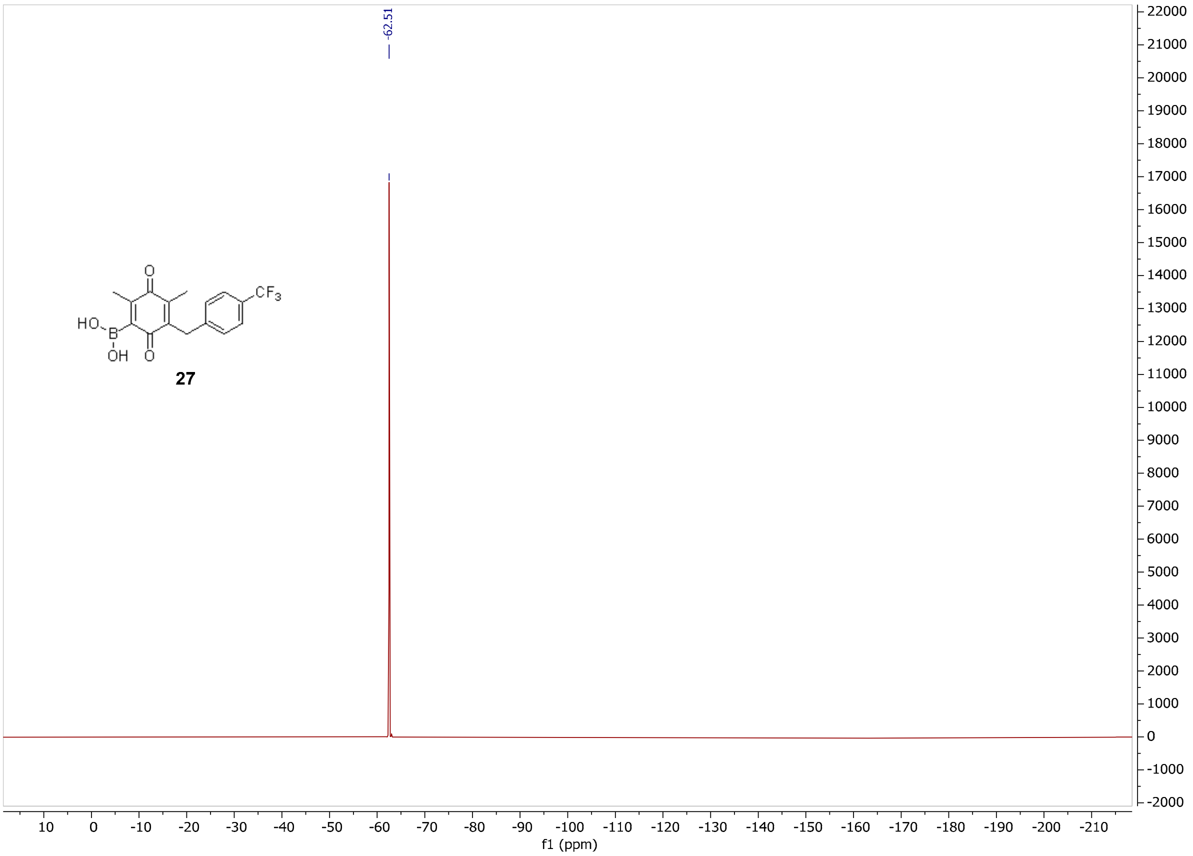

^{13}C {^{1}H} NMR spectra of 27.

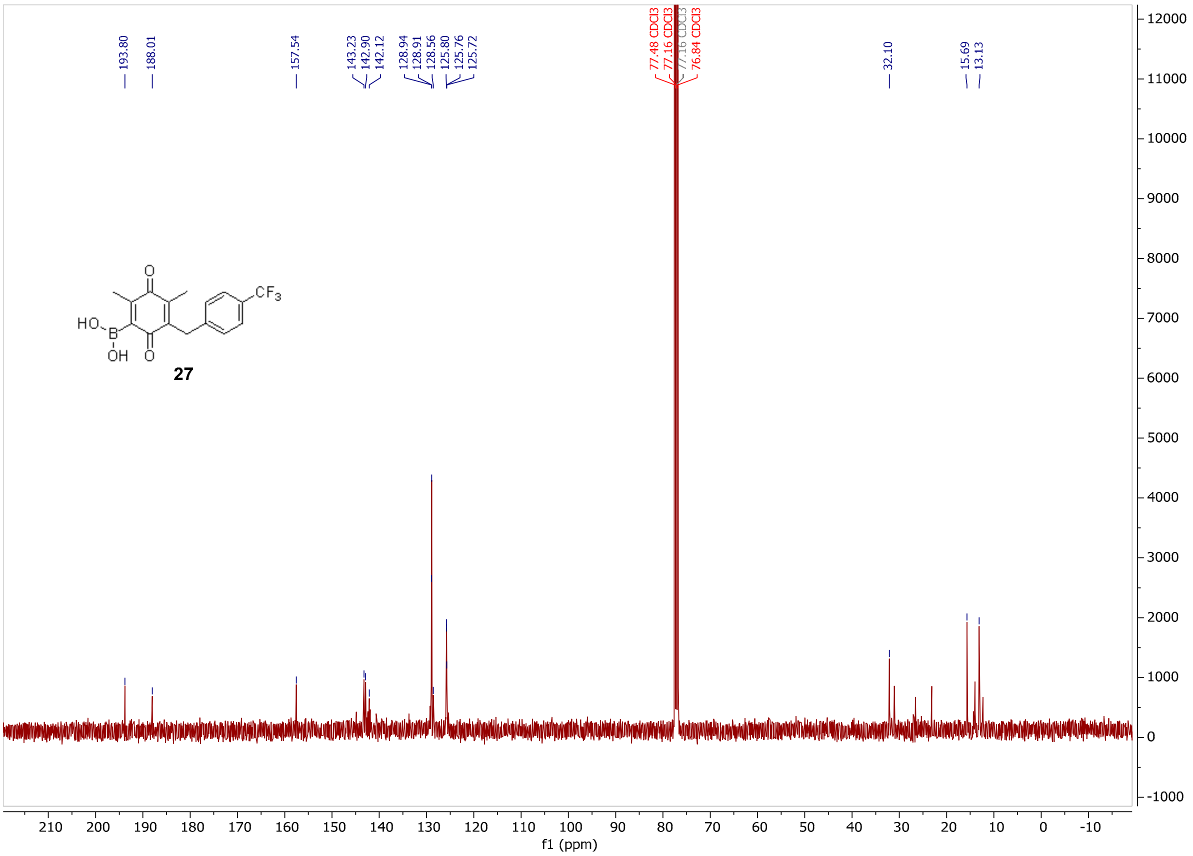

Supplement: Supplementary file 1 [file molecules-29-05268-s001.zip › molecules-3212870-supplementary.pdf]
